# Indoor-air purification by photoelectrochemical oxidation mitigates allergic airway responses to aerosolized cat dander in a murine model

**DOI:** 10.1038/s41598-023-38155-0

**Published:** 2023-07-06

**Authors:** Dinesh Devadoss, Kerri Surbaugh, Marko Manevski, Chatura Wickramaratne, Dale Chaput, Arianne Chung, Francisco de Leon, Hitendra S. Chand, Jaspreet S. Dhau

**Affiliations:** 1 Department of Immunology and Nano-Medicine, Herbert Wertheim College of Medicine, Florida International University, 11200 SW 8th St, Miami, FL 33199 USA; 2 Research and Development, Molekule Group, Inc., 3802 Spectrum Blvd, Tampa, FL 33612 USA; 3 Department of Cell Biology, Microbiology and Molecular Biology, University of South Florida, Tampa, FL 33612 USA

**Keywords:** Biochemistry, Immunology, Environmental sciences, Chemistry, Engineering, Materials science

## Abstract

Portable air purifiers help improve indoor air quality by neutralizing allergens, including animal dander proteins. However, there are limited in-vivo models to assess the efficacy of these devices. Here, we developed a novel animal model of experimental asthma using aerosolized cat dander extract (CDE) exposure and compared the efficacy of select air purification technologies. Mice were exposed to CDE aerosols for 6 weeks in separate custom-built whole-body exposure chambers equipped with either a photoelectrochemical oxidative (PECO) Molekule filtration device (PFD) or a HEPA-assisted air filtration device (HFD) along with positive (a device with no filtration capability) and negative controls. Compared to the positive control group, the CDE-induced airway resistance, and plasma IgE and IL-13 levels were significantly reduced in both air purifier groups. However, PFD mice showed a better attenuation of lung tissue mucous hyperplasia and eosinophilia than HFD and positive control mice, indicating a better efficacy in managing CDE-induced allergic responses. Cat dander protein destruction was evaluated by LCMS proteomic analysis, which revealed the degradation of 2731 unique peptides on PECO media in 1 h. Thus, allergen protein destruction on filtration media enhances air purifier efficacy that could provide relief from allergy responses compared to traditional HEPA-based filtration alone.

## Introduction

One critical aspect of managing allergic asthma is maintaining a clean indoor air environment, and avoiding exposure of patients to allergens and insults, especially when they are sensitized to the consistently present indoor allergens that trigger the asthmatic response. The association between indoor allergens and asthma morbidity, especially in the development of childhood asthma, has been established previously^1^. Some common allergens include house dust mites, cockroaches, pollens, mold spores, and metabolites; however, pet dander is a prevalent household allergen that is frequently overlooked. In particular, cat dander is the most prominent indoor allergen, and in that Felis domesticus 1 (Fel d1) is well-proven and the dominant allergen component of cat dander^2–4^. Fel d1 is a secretoglobin protein complex known to induce a Th2 immune response, and approximately 50% of asthmatic patients have been shown to have IgE-mediated allergic responses upon exposure to Fel d1. Fel d1 is a highly prevalent component in cat sebaceous glands, from which it is secreted onto the fur and skin and is also present in the cat salivary glands^5–7^.

Improving indoor air quality is paramount for successfully managing allergic responses to airborne allergens and improving asthmatic patients’ overall quality of life. However, integrating an air purification regime has yet to be considered for asthma treatment and/or prevention options. Although the current treatment regimens, including the allergen-specific immunotherapy, have shown some improvement^8–11^, technological advances in inactivating the environmental allergens should also be the primary focus of efforts to help reduce/limit the asthmatic exacerbations. Non-invasive, environmental, and physical methods to avoid sensitization, including the use of portable air purifiers, are crucial in eliminating airborne allergens. However, the efficiency of aeroallergen clearance and potential health benefits may vary based on the types of environments, filtration technologies, and the devices being used.

Air purification is a marathon effort rather than solely a sprint where how quickly particulate matters are removed from the air is considered important. There is more to air purification than solely demonstrating the removal or reduction in pollutant levels below the standard detection limits. However, living beings, including humans and animals are significantly more sensitive to changes in pollutant levels than any currently used analytical instruments. Therefore, continuous efforts are needed to understand the impact of air purification on respiratory health so that it can be judiciously integrated in everyday life. The current study endeavors to develop a method to evaluate air purification technologies, especially when standard analytical methods had limited success in establishing a conclusive link between health benefits and air quality parameters.

Air is a multi-component mixture of gases, particles, and liquid droplets known as aerosols (Fig. S1). Allergen particles are one component of air which are understudied. Allergen particles in the air are generally considered intact and immobilized on dust particles of different size ranges. Therefore, all the test methods to evaluate the efficacies of air purifiers are based on particle removal approaches and do not address the concerns from the presence of free allergens and allergen proteins in the air. Free allergens and allergen proteins represent allergen particles in the nanometer range that are not immobilized on the bigger dust particles. To the best of our knowledge, no analytical instrumentation method is available which can determine the presence of free-allergen proteins, segregated from the dust-immobilized particles, and in that case, evaluate the efficacies of air purification technologies and devices. Accordingly, we posit that the elimination of free-allergen proteins from the air is critical for reducing sensitization and asthmatic exacerbations.

A recently developed PECO air purification technology (Fig. S2) captures and destroys (oxidizes) organic pollutants and allows the elimination of particles 1 × 10^3^ times smaller than particles removed by mere physical filtration-based air purification technologies^12^. The organic pollutants, include Semi-volatile, and Volatile Organic Compounds (sVOCs, and VOCs, respectively), pathogenic microbes, and allergen particles. PECO-assisted air filtration device (PFD) (Molekule Air Mini+) have been approved as Class II medical devices by the US Food and Drug Administration (FDA) due to their efficiency and potential to eliminate pathogenic bacteria and viruses^13^. In a pilot-scale clinical study, these Molekule air purifiers were shown to reduce the length of hospitalization, rates of intubation, and the need for nebulizers and non-invasive intervention in pediatric patients hospitalized due to respiratory distress^14^. However, further study is required to investigate the full potential and limitations, and the utility of this novel technology in human-health-related fields.

The design of the present study was based on the following questions.

1. Does standard method of determining particulate levels in air sufficient to evaluate the efficacy of air purifier units?
2. Can the presence of Fel d1 levels that are below the detection limit of an analytical method cause allergic responses in an experimental mouse model?
3. Does destruction of allergen proteins on the PFD impact the performance of the air purifier unit, beyond the physical filtration parameters?

In this study, a small preclinical animal model was employed to investigate the above-mentioned queries and compare the efficacy of a novel PECO-assisted portable purification system (with PFD) to a traditional HEPA filter-based system (with HFD). Adult C57BL/6J mice were exposed to aerosolized CDE in whole body chambers to mimic the conditions resembling indoor household allergen exposure, and the effects of the air purification systems were tested to quantify their efficiency in minimizing the CDE-induced asthmatic responses. Our aerosolized CDE exposure model induced an asthmatic response in mice and effectively mimicked the household pet dander allergen exposure conditions. The lung function parameters, broncho-alveolar lavage fluid (BALF) cytokines and cell differential, lung tissue histology, and airway epithelial remodeling as well as the plasma cytokine levels were analyzed to compare the effects of air filtration on CDE-induced allergic inflammatory responses. Overall, our study suggests that the PFD and its destruction technology is more effective in preventing the CDE-induced allergic asthma symptoms and exacerbations in a mouse model. This effect could primarily be due to the efficient oxidative inactivation of allergen proteins as verified by our LC–MS proteomic analysis studies. Thus, the novel PFD destroys the biological activity of aeroallergen proteins and use of such devices could provide relief from debilitating allergic exacerbations.

## Results

### Exposure conditions

Animals in all the chambers (NC—Negative Control; PC—Positive Control; PFD—PECO-assisted air filtration device; HFD—HEPA-assisted air filtration device) were exposed to similar environmental conditions as reflected by the average temperature and humidity recorded during the study (Table 1). The average temperature in all the chambers ranged between 20.3 and 21.7 °C (0.1) with the humidity levels ranging from 49.9 to 56.7% during the study.

**Table 1:** Average chamber temperatures and humidity percentages during the study period.

Chamber	Initial		End Temp (°C)		End humidity (%)	
	Temp (°C)	Humidity (%)	Min	Max	Min	max
NC	21.36	55.38	21.12	21.73	53.08	56.92
PC	21.11	54.92	21.06	21.45	53.31	56.62
PFD	20.37	55.54	20.32	21.02	51.62	56.69
HFD	20.85	53.08	20.82	21.61	49.92	54.54

*NC* negative control, *PC* positive control, *PFD* PECO-assisted air filtration device, *HFD* HEPA-assisted air filtration device.

### CDE aerosol concentrations

The analysis of the cat dander allergen protein Fel d1 in all chambers’ air samples were performed by Indoor Technologies Inc. There were comparable levels of CDE aerosol generated in each of the chambers (PC, PFD, and HFD chambers) throughout the six-weeks of study with an average Fel d1 levels achieved at 60.05, 64.74, and 59.62 ng per 2 L of chamber air sample (Table 2). Most importantly, the air samples collected pre- and post-air-filtration shows that both the PECO-assisted Air Mini+ and the HEPA filter-assisted air purifier devices were able to suppress the Fel d1 to below the detection limit levels in the each of the chambers compared to the positive control chamber where there was still 50% of the initial Fel d1 aerosols was present in the air sample.

**Table 2:** The Fel d1 aerosol concentration in PC, PFD, and HFD chambers at pre and post air-filtration determined as ng per 2L of chamber air sample.

	PC Chamber #2		PFD Chamber #3		HFD Chamber #4	
	Pre	Post	Pre	Post	Pre	Post
Wk1	55.08	22.60	70.00	BDL	63.26	BDL
Wk2	76.94	43.01	53.93	BDL	47.07	BDL
Wk3	97.21	44.97	54.18	BDL	50.55	BDL
Wk4	46.13	27.34	82.05	0.08	71.51	BDL
Wk5	55.91	46.15	65.78	BDL	71.45	BDL
Wk6	29.02	17.36	62.54	BDL	53.89	BDL
Mean	60.05	33.57	64.74	0.08	59.62	BDL
± SEM	9.77	5.16	4.33	BDL	4.35	BDL

*BDL* below detection limit, *NC* negative control, *PC* positive control, *PFD* PECO-assisted air filtration device, *HFD* HEPA-assisted air filtration device.

### Mice body weights and lung function analyses

**Figure 1:**
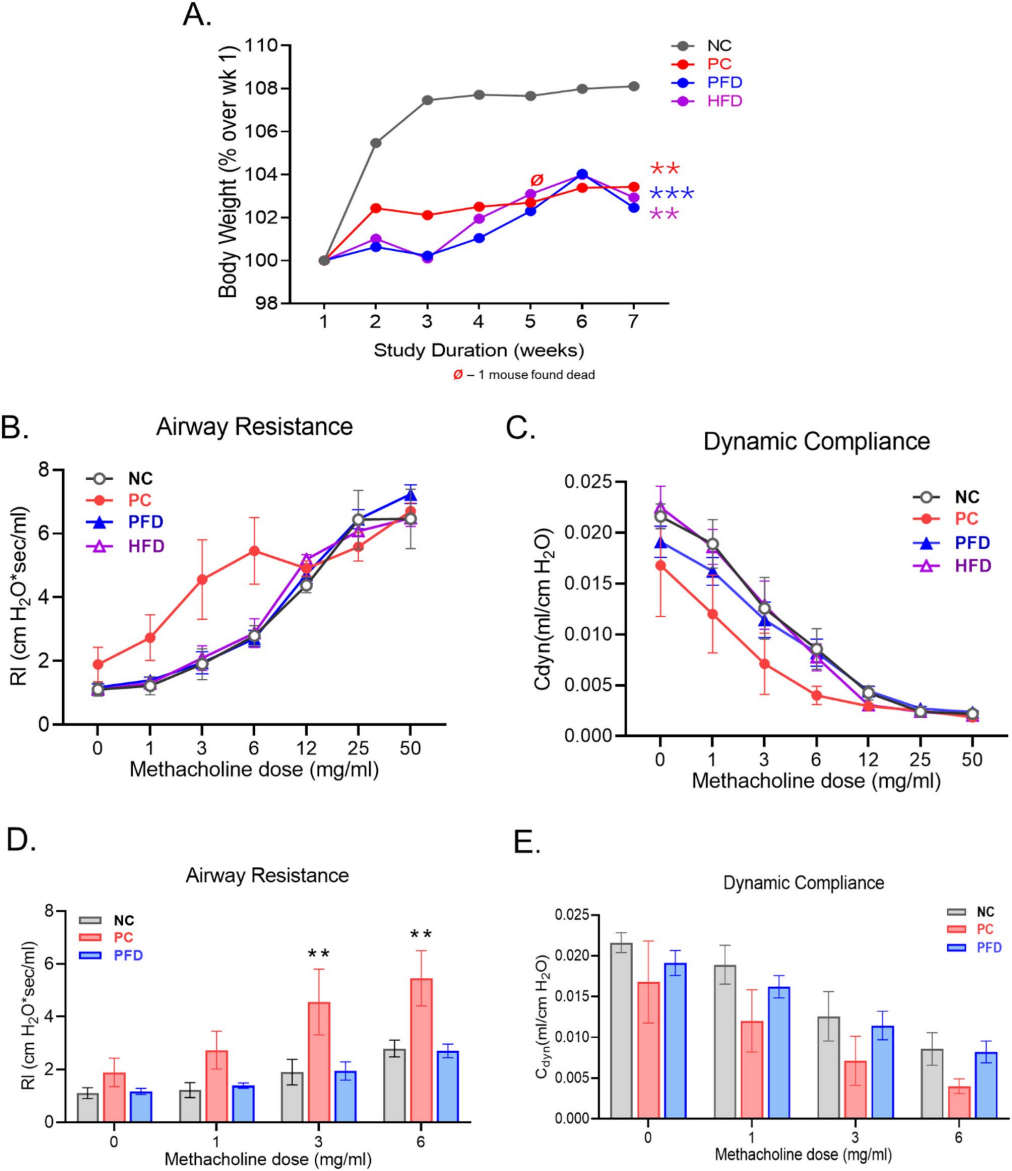
(**A**) Changes in the mice body weights in each group during the study shown as the percentage change over average initial body weight for each group. Data shown as mean ± SEM and analyzed by ANOVA (n = 10/gp); **p < 0.01; ***p < 0.001 compared to NC group. Lung function parameters analyzed following CDE-exposure and air filtration in each mice group. The (**B**) airway resistance (R_L_) and the (**C**) dynamic compliance (C_dyn_) of anesthetized mice from each group were determined following the increasing dose of methacholine (MCh) (0, 1, 3, 6, 12, 25 and 50 mg/mL) aerosol challenges via inhalation. The histograms showing individual group (**D**) R_L_ and (**E**) C_dyn_ values for mice challenged with 0, 1, 3, and 6 mg/mL of methacholine. Data shown as mean ± SEM (n = 5/gp) and analyzed by 2-way ANOVA; **p < 0.01.

Animals in the study weighed between 26 and 32 g at the beginning of the study with an average body weight in each group of approximately 30 g. The changes in the body weights were calculated as the percentage of the initial body weight for each mouse and average change was plotted for each group (Fig. 1A). Animals in the room air gained around 8% of body weight during the study whereas the mice exposed to CDE aerosol gained less weight (2%) with no significant difference among the PC, PFD, and HFD mice. Next, at the end of study, five animals from each group were randomly selected and were subjected to pulmonary function analysis to determine changes in airway hyperreactivity following methacholine (Mch) bronchoprovocation. There was a significant increase in airway resistance (R_L_) in PC group of mice at 3 and 6 mg/mL of Mch challenges compared to the NC group (Fig. 1B,D). Both the PFD and HFD mice airways were not sensitive to Mch challenges with changes in lung function parameters not distinctive from the NC group indicating that air-filtration by both PFD and HFD has functionally suppressed the CDE allergen levels. There were no significant changes observed in the dynamic compliance (C_dyn_) of mice airways for any of the groups tested (Fig. 1C,E).

### BALF cells and airway eosinophilia

**Figure 2:**
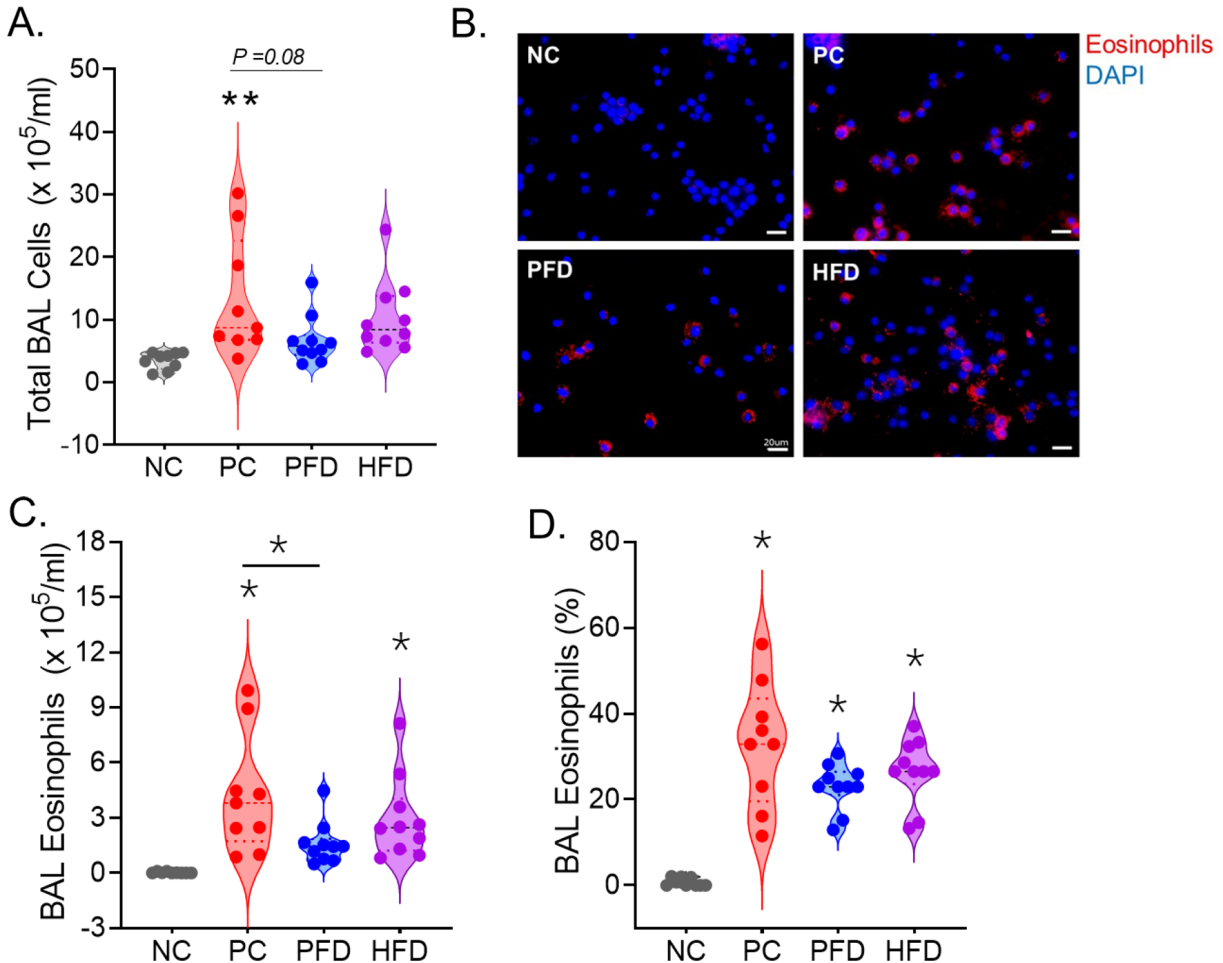
CDE aerosol induced airway eosinophilia is suppressed by PFD. (**A**) Violin plot analysis of total BALF cells obtained from each group where each dot represents individual mouse per group. (**B**) Representative micrographs showing immunostained eosinophils (shown in red) in BALF cytospins for each group with nuclei stained with DAPI (shown in blue), scale bar—10 µm. Quantitation of (**C**) total eosinophils and (**D**) eosinophil percentage per ms in each group. Data analyzed by ANOVA (n = 10/gp except for PC, n = 9); *p < 0.05, **p<0.01.

Among the BALF cells collected, there were on an average 3.53 × 10^5^ cells/mL in the NC group. The CDE allergen exposure induced the cellularity in BALF by fourfold yielding 12.05 × 10^5^ cells/mL in PC group. There was a noticeable attenuation (*p* value = 0.08) of the BALF cell recruitment in PFD animals with an average of 6.14 × 10^5^ cells/mL (Fig. 2A). Whereas the HFD mice only showed a trend toward lower BALF cell counts with an average of 9.44 × 10^5^ cells/mL. Most of this cell infiltration was due to eosinophil (Eos) recruitment as determined by immunostaining of BALF cytospins (Fig. 2B) and quantification of total Eos (Fig. 2C) as well as of total BALF cell percentage per mouse (Fig. 2D). Eos accounted for around 32% of BALF cells in PC group with 3.96 × 10^5^ Eos/mL that were significantly suppressed in the PFD mice.

### Plasma and lavage IgE and IL-13 levels

**Figure 3:**
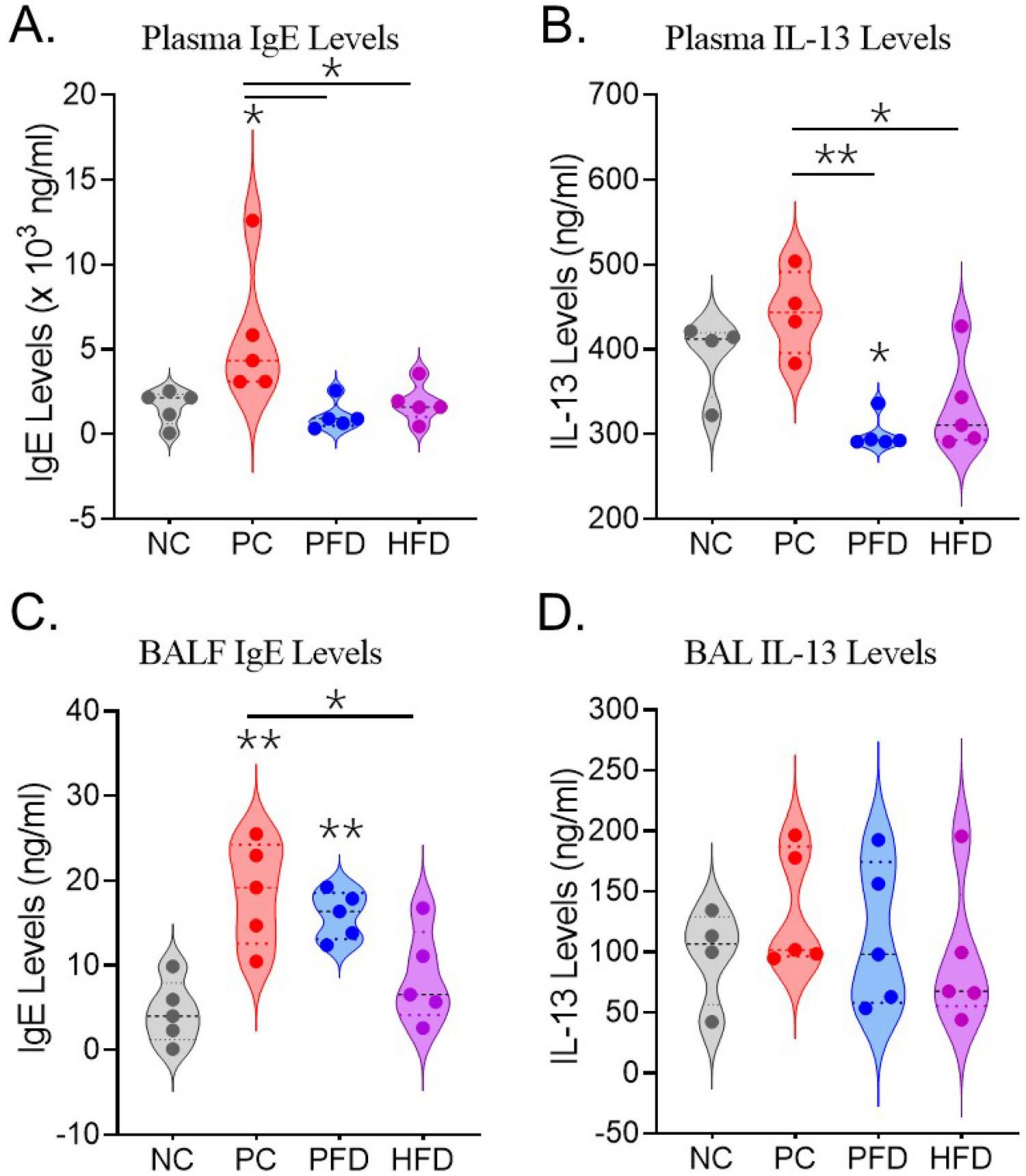
Quantitation of plasma (**A**) IgE and (**B**) IL-13, and BALF (**C**) IgE and (**D**) IL-13 levels in mice from each group as determined by ELISA. Data analyzed by ANOVA (n = 5/gp); *p < 0.05; **p < 0.01.

Plasma and lavage fluid collected from each mouse were assessed for total IgE levels and IL-13 cytokine levels using respective ELISAs. There was a significant increase in plasma IgE levels that were attenuated in the PFD and HFD groups (Fig. 3A). A similar significant suppression was observed in the plasma IL-13 levels of PFD and HFD groups that was induced in the PC group (Fig. 3B). Analysis of BALF IgE levels also showed a similar trend where PFD and HFD groups showed a better attenuation of CDE-induced IgE levels (Fig. 3C) but there was no difference in the BALF IL-13 levels among the mice of all the groups analyzed (Fig. 3D).

### Airway mucous metaplasia and peribronchial eosinophilia

**Figure 4:**
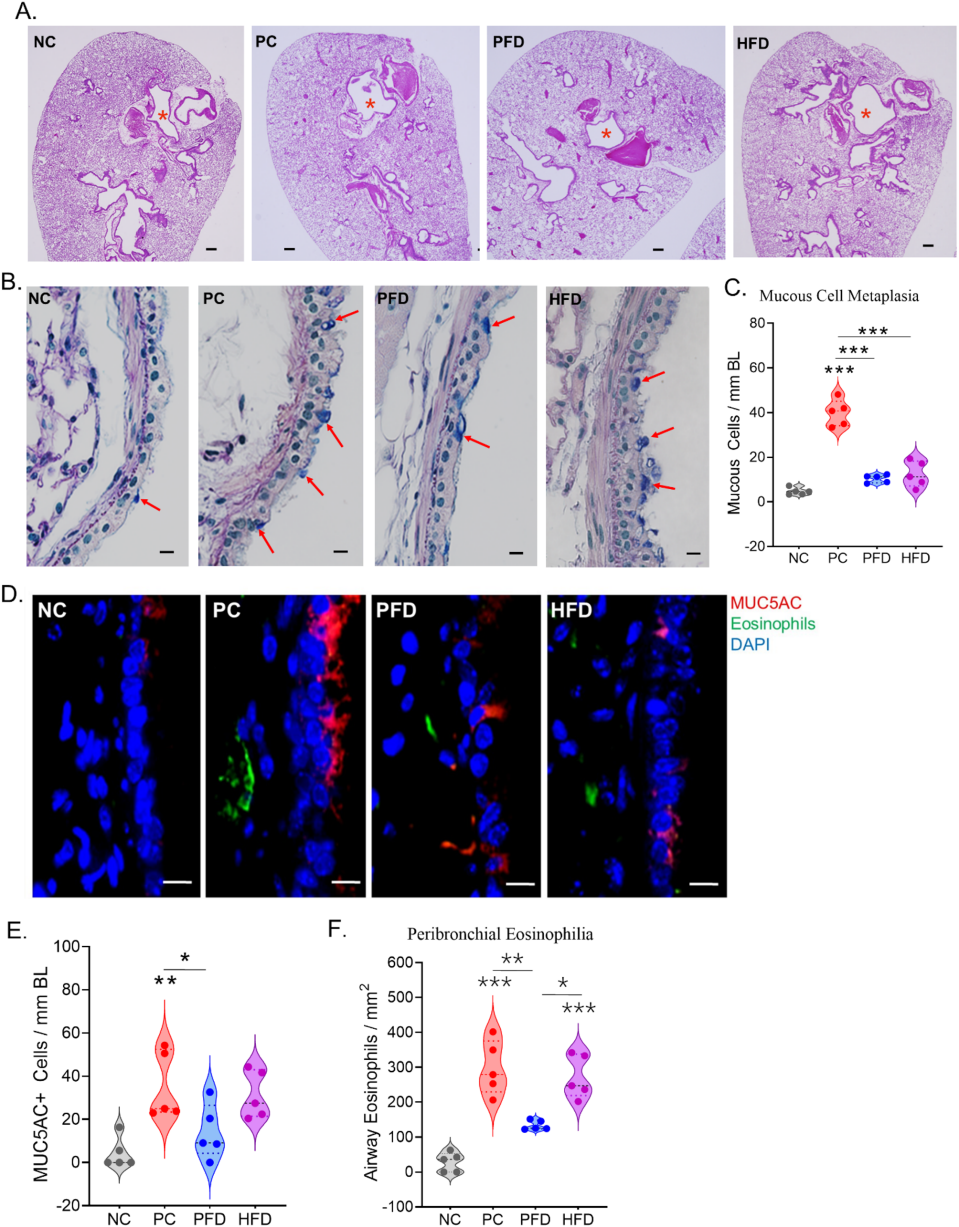
The airway mucous cell metaplasia and peribronchial eosinophilia caused by CDE aerosol exposure is mitigated by PFD. Representative micrographs of mouse lung tissue from each group showing gross morphology and airway mucous metaplasia. (**A**) Low magnification images of whole lung section stained with H&E showing the bronchial airways (marked with a red asterisk), scale—200 µm. (**B**) A high magnification image of lung axial airways stained with AB/PAS showing the AB + mucin glycoprotein marking the mucous/goblet cells (marked with red arrows), scale—10 µm. (**C**) The Quantitation of mucous cells per mm of Basal lamina (BL). (**D**) Representative micrographs showing airway Muc5ac + mucous/goblet cells (shown in red) and the eosinophils (shown in green) with nuclei stained with DAPI (shown in blue), scale bar—10 µm. Quantitation of (**E**) Muc5ac + cells per mm of BL and (**F**) Eosinophils per mm^2^ area in each group of mice. Data shown as mean ± SEM and analyzed by ANOVA (n = 5/gp). *p < 0.05; **p < 0.01; ***p < 0.001.

There was no significant difference in gross lung tissue histopathology in any of the groups in terms of cellularity around airways, blood vessels, or in parenchyma (Fig. 4A). However, the axial airways showed increased mucous metaplasia in PC mice as shown in Fig. 4B and the PFD and HFD group showed some attenuation of the mucous cell numbers per mm BL (Fig. 4C). The changes in mucous cell metaplasia were further confirmed by immunostaining of serial sections with antibody to Muc5ac mucin that showed significant attenuation in PFD mice than the HFD mice (Fig. 4D,E). The sections were also co-stained for eosinophils that marked a significant peribronchial eosinophilia in PC mice compared to NC mice and the PFD significantly attenuated this increased eosinophil recruitment whereas HFD group mice failed to show any change from the PC group (Fig. 4D,F).

### PECO-assisted protein destruction mechanism

To understand the action of PECO on proteins, filter swatch analysis was performed using electrophoresis technique with allergen, and non-allergen proteins such as Fel d1 as well as *A. niger* protein, and bovine serum albumin (BSA), respectively. The protocol was tested on two types of PECO media (PECO 1.0 and PECO 2.0), Carbon, and HEPA filter media, and the results were analyzed to compare the performance. PECO 1.0 is the legacy media and PECO 2.0 is an upgrade which is currently being used in Molekule Air purifier units.

**Figure 5:**
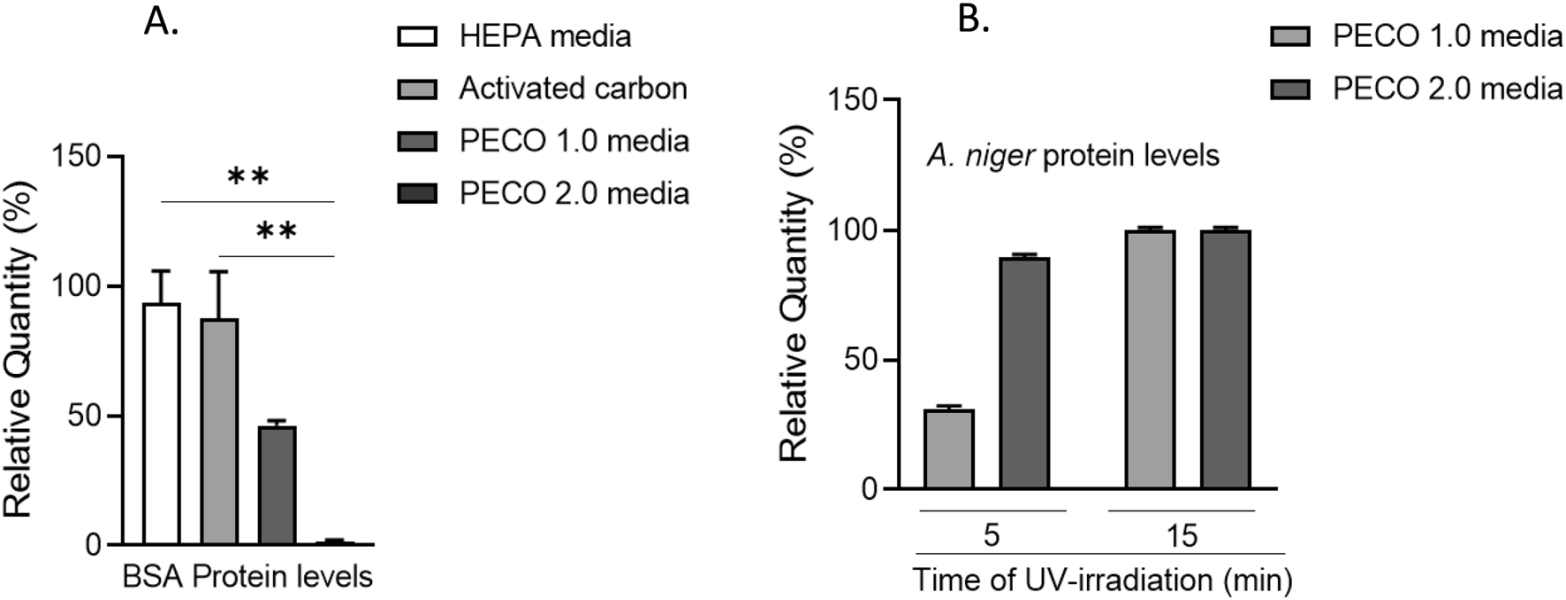
Filter swatch analysis showing (**A**) percentage of BSA protein degradation with 30 min UV-A exposure and, (**B**) percentage of *A. niger* protein extract degradation in 5 and 15 min of UV-A exposure. Data shown as mean ± SEM and analyzed by ANOVA (n = 3); **p < 0.01.

As evident from the graph in Fig. 5A and Fig. S7, 98.6% (± 0.69%) of BSA degraded on the PECO 2.0 media (currently used in Molekule Air Purifiers) and 53.8% (± 1.88%) on PECO 1.0 media (legacy PECO filter) in 30 min exposure to UV-A, whereas only 12.1% (± 18.13%) and 7.0% (± 12.13%) got denatured or remained adsorbed on the carbon, and HEPA filters, respectively. Next, *A. niger* protein extract was exposed to 5 and 15 min of UV-A irradiation. After 5 min of UV-A irradiation (Fig. S8), *A. niger* protein extract degraded 69% (± 1.25%) and 89.6% (± 1.0%) on the PECO 1.0 and 2.0 media, respectively. After 15 min of UV-A irradiation (Fig. S9), *A. niger* protein extract degraded 100% (± 1.0%) on the PECO 1.0 and 2.0 media (Fig. 5B). Similarly, as determined by SDS PAGE analysis, pure feld1 degraded completely on PECO 1.0 and 2.0 media in 30 min of exposure to UV-A (Fig. S10).

### CDE proteomics analyses by LC–MS

**Figure 6:**
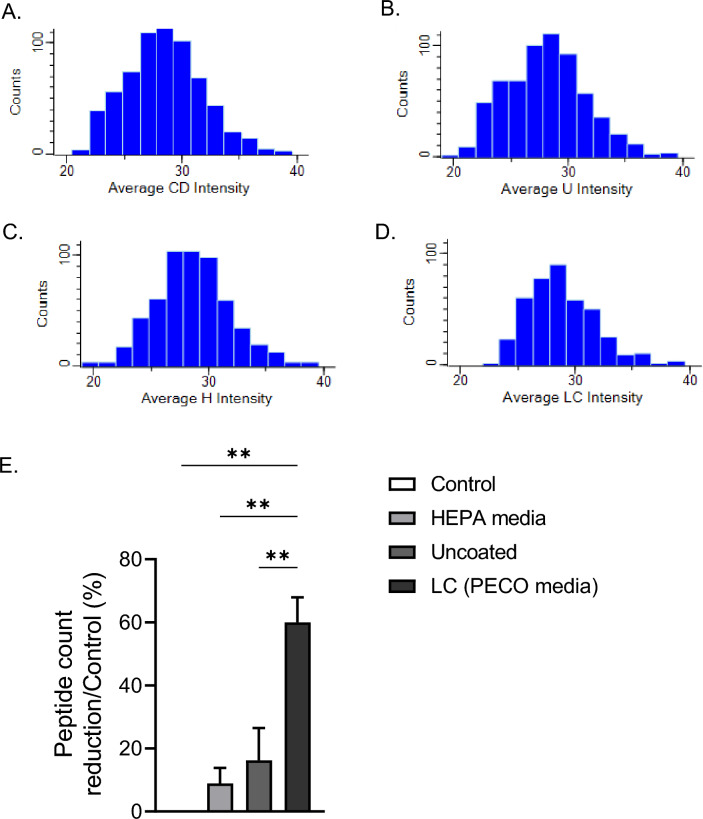
The PECO 2.0 demonstrated a reduced number of proteins as compared to the uncoated and HEPA filter media swatches contaminated with cat dander extract. Histograms showing the number of proteins (Counts) and their Log2 Intensity values for (**A**) cat dander/Control, (**B**) uncoated filter media (U), (**C**) HEPA media (H), and (**D**) PECO 2.0 filter media (LC) contaminated with cat dander. (**E**) The percentage of protein degradation by normalized with control/whole solution of cat dander. Data shown as mean ± SEM and analyzed by ANOVA (n = 3); **p < 0.01.

Protein analysis from crude CDE by SDS PAGE analysis was cumbersome as no distinct bands were observed on the electrophoresis plate. Therefore, we explored S-Trap Mini Ultra-High Recovery Protocol and liquid chromatography–mass spectrometry to develop a complete profile of the proteins from the cat dander extract and evaluated the destruction of CDE proteins on the PECO filtration media. Perseus software was used to annotate allergens from the UniProt database for *Felis silvestris catus*. The protein degradation is measured as a percent decrease between the LFQ intensities of whole solution of cat dander (Fig. 6A–D). A total of 4492 unique peptides were identified by LCMS analysis for the control sample (CDE solution) and out of these 2731 (61%) peptides were completely degraded by PECO 2.0 media when exposed to UV-A for one hour. On the other hand, HEPA swatches showed < 8% peptide reduction, which is mostly due to inefficiency in completely extracting the trapped proteins on the swatch samples (Fig. 6E). These data support the animal model study and further illustrate the PECO technology as an additional management tool for allergy sufferers.

## Discussion

The present study shows that a developed mouse model of chronic inhalational exposure of aerosolized CDE in an indoor full-body exposure chamber induces airway hyperreactivity and an asthma-like response. Specifically, airway resistance, eosinophil levels in BALF, blood plasma IgE, and cytokine IL-13 levels were significantly higher in mice exposed to CDE in the PC group, with an air purifier fitted with a sham air filter. Histopathology showed mucus metaplasia in conducting airways of these animals. Interestingly, although both PFD and HFD eliminate the Fel d1 levels below the detection level in the test-chamber and help attenuate the effects observed in the PC group, we found that the PECO filter works more efficiently than the HEPA filter.

Salient clinical phenotypes of allergic asthma response include eosinophilic infiltration, airway restriction, and mucus/phlegm hyperexpression in the respiratory tract. The inflammatory response following allergen exposure/sensitization involves an elevated secretion of IL-4, IL-5, IL-13, granulocyte–macrophage colony-stimulating factor (GM-CSF), and additional cytokines/chemokines, resulting in the recruitment and activation of other immune cells^15–18^. A switch in the circulatory levels of immunoglobulin E^19^, another classical immunophenotype, further aggravates the inflammatory response leading to the asthmatic exacerbation. Accordingly, in addition to conventional therapies, more effective therapeutics are currently being developed by targeting these proinflammatory molecules. For example, an anti-IL-5 antibody-based therapy is showing some potential against allergic asthmatic responses in the ongoing clinical trials^19,20^. However, these therapies including those targeting IgEs, eosinophilia, or airway hyperplasia have shown limited success in preclinical and clinical studies^16,17,21,22^. Thus, avoiding allergen exposure and/or sensitization altogether is a critical preventative measure for patients with allergic asthma whereby maintaining an environment free of active allergens. This study suggests that novel PECO-assisted air filtration may provide an opportunity to fulfil this need.

It has been reported that patients suffering from allergic asthma could get relief with the use of air purifiers, however, more in-depth investigations are needed to improve the overall impact^23^. Study results have often been inconclusive primarily due to the use of inappropriate experimental models or study design. For example, a study investigated the effect of using HEPA-filter utilizing air purifiers in classrooms on students with active asthma and found that school-wide use of HEPA filter purifiers did not significantly reduce symptoms in patients suffering from active asthma^24^. However, the authors suggested that additional examination and study is necessary to better understand the relation between air purification and asthma relief. Interestingly, modifications of HEPA filters such as coating or chemical modifications have also been proposed to improve their efficiency in capturing pathogen particles^25^. It is evident here that there is more to air purification than mere physical filtration of the particles.

PFDs are proposed to be novel improvements to the current filtration technologies available on the market. They offer to provide additional protection-layer by a nano-coated filter that oxidizes organic matter to benign species, water, and carbon dioxide. This novel technology allows for exceptional capability to improve upon the filtration technologies in an era where a need for successful pathogen elimination by filtration elements has not been comprehensively met^26^. Here, we show that there may be strong effectivity of PECO-based air filtration to attenuate the effects of a CDE-mediated asthmatic model in a rodent full body exposure setting.

### Impact of PECO filtration and mechanism of protein degradation on PECO media

HEPA filtration works through capture mechanism involving impaction, diffusion, and interception with an efficiency of 99.97%, i.e., only three particles may pass through the filter out of 10,000 particles. PECO works, in addition to the capture mechanism, through collision, adsorption, and oxidation reaction mechanism. Allergen protein particles may pass through the HEPA filter without any damage and with a capability to cause allergic reactions. On the other hand, allergen proteins may get denatured due to its reaction with reactive oxygen species (ROS) on its passage through the PECO filter. The filter technology of Molekule Air-Mini is advanced using nano-catalytic TiO_2_ and low-energy UV-A light, which supports a variety of reactions (Eqs. 1 to 7) on the filter surface generating sufficient short-lived ROS, including hydroxyl radicals, peroxide and superoxide ions, to oxidize allergen proteins efficiently to benign products such as CO_2_, N_2_, H_2_O etc. PECO reaction rate optimization has been achieved by high PECO filtration surface area, optimum air flow characteristics, and UVA-LED light intensity.

1 $${\text{TiO}}_{{2}} \left( {{\text{hv}}} \right) \, \to {\text{ TiO}}_{{2}} \left( {{\text{e}}^{ - } + {\text{ h}}^{ + } } \right),$$

2 $${\text{TiO}}_{{2}} \left( {{\text{e}}^{ - } + {\text{ h}}^{ + } } \right) \, + {\text{Feld1 }} \to {\text{ TiO}}_{{2}} + {\text{ Feld1}} _{{\text{(ads)}}} \to {\text{ Degraded protein products,}}$$

3 $${\text{TiO}}_{{2}} \left( {{\text{h}}^{ + } } \right) \, + {\text{ H}}_{{2}} {\text{O }} \to {\text{ TiO}}_{{2}} + {\text{ HO}}^{ \cdot } \, + {\text{ H}}^{ + } ,$$

4 $${\text{TiO}}_{{2}} \left( {{\text{h}}^{ + } } \right) \, + {\text{ OH}}^{ - } \, \to {\text{ TiO}}_{{2}} + {\text{ OH}}^{ \cdot } ,$$

5 $${\text{TiO}}_{{2}} \left( {{\text{e}} - } \right) \, + {\text{ O}}_{{2}} \to {\text{ TiO}}_{{2}} + {\text{ O}}^{{{2} \cdot - }} ,$$

6 $${\text{O}}^{{{2} \cdot - }} + {\text{ H}}^{ + } \to {\text{ HO}}_{{2}}^{ \cdot } ,$$

7 $${\text{Feld1}}\, + \,{\text{ROS}}\, \to \,{\text{Degraded protein products }}\left( {{\text{CO}}_{{2}} ,{\text{ H}}_{{2}} {\text{O}},{\text{ N}}_{{2}} {\text{etc}}.} \right).$$

### Conclusion and limitations

Based the reported study on animal model and subsequently proteomic analysis, the following can be concluded:

1. Standard methods of determining particulate level alone are not sufficient to evaluate the efficacy of air purifier units, especially related to health concerns.
2. The presence of Fel d1 at below the detection limit level in air could cause allergic responses in mice.
3. Photoelectrochemical oxidation-based air purification technology is an efficient tool for degradation of allergens in the air.
4. Destruction of allergen proteins on the PECO filter impacts the performance of the air purifier unit, beyond the physical filtration parameters and provide an additional layer of protection.

The primary limitation of this study is the animal numbers used per group. We utilized 10 animals per group, and for some analysis we used only five animals. So, further studies are needed using a larger number of animals to help establish the efficacy of the air filtration devices and help design the preclinical and clinical trials needed to expedite the clinical use of these devices. To summarize, we have shown here that the potential of PFDs show promise and needs further exploration, and their benefit in allowing patients suffering from allergic asthma to avoid sensitization, combined with their portable design makes them excellent candidates for further development. Further large-scale preclinical studies and technology developments are needed to help establish the clinical efficacy of such filtration devices for human use.

Controlled studies are urgently needed to investigate the effects of portable air purifiers on incidence of respiratory infections^27^. Lastly, the evaluation of a filter lifespan and the replacement needs are also to be investigated in these experimental models. In this regard, a dust loading level modeling has been proposed as a method of monitoring the need for filter replacement and shows potential for further development^28^.

## Materials and methods

### Animals and study groups

Male pathogen-free wild type (WT) C57BL/6J mice of 18-weeks age were purchased from The Jackson Laboratory (Bar Harbor, ME) and were quarantined for 10 days under pathogen-free conditions. All experiments were approved by the Institutional Animal Care and Use Committee (IACUC) and were conducted at Florida International University (FIU), a facility approved by the Association for the Assessment and Accreditation for Laboratory Animal Care International. Briefly, after the quarantine, 40 mice (n = 10/group) were then acclimatized to the exposure chambers for 5 days before the study. Mice were then weighed, randomized, and divided into four groups as shown in Table 3. Group 1 or negative control (NC) group mice were placed in a chamber with the ‘sham’ air-filter (portable air filter with no active filtration unit) to control for the effects of noise, heat, and vibration of the filtration units, and mice were exposed to room air only. The other three groups were exposed to aerosolized CDE (Greer Labs, Lenoir, NC) on alternate days for 6 weeks. Group 2 or positive control (PC) group mice had a sham air-filter. Group 3 or PFD group mice had PECO-assisted filtration unit in the chamber. Group 4 or HFD group mice had the traditional HEPA filter unit in the chamber.

**Table 3:** Summary of study groups with exposure conditions and the type of air-filters used.

Group	No. of mice	Exposure/challenge	Condition	I.D.
1	10 (5 × 2 Cages)	Room air	Sham air filter	Negative control (NC)
2	10 (5 × 2 Cages)	Cat dander extract	Sham air filter	Positive control (PC)
3	10 (5 × 2 Cages)	Cat dander extract	Photoelectrochemical oxidative Molekule Air Mini + device	PECO filter device (PFD)
4	10 (5 × 2 Cages)	Cat dander extract	HEPA-assisted air purification device	HEPA filter device (HFD)

### Exposure chambers set-up, CDE aerosolization and animal exposures

The detailed step-by-step chamber set-up, CDE aerosolization, air sampling and animal exposure procedures were presented in Supplementary Information under Protocol S1. In here briefly, the whole-body inhalation exposure chambers were configured and custom-built to efficiently manage the exposure schedule (Fig. S3). The PC, PFD, and HFD group mice were exposed to CDE aerosols in their own chambers and NC group mice were exposed to room air with the sham air filter in a separate room to avoid cross-contamination. To ensure full exposure, the lid on the filter cage was removed in all chambers, allowing the animals to inhale the conditioned ambient environment above. The temperature and humidity in all chambers were monitored during the exposures. Mice in PC, PFD and HFD group were exposed to CDE aerosols for 1 h/day and 3 days/week for 6 weeks as outlined in Fig. S4. The tidal volume of mouse breath per minute is 1.46 mL air/gram of mouse mass^29,30^. A mass balance equation with variables specific to our chamber and experiment was used to determine the starting concentration of CDE/L air in the exposure chambers. Figure S5 in the Supplementary Information demonstrates natural decay of CDE level inside the chamber during 1 h duration. CDE was nebulized using a syringe pump and a Blaustein Atomizing Module (BLAM, CH Technologies USA, Westwood, NJ) to achieve CDE deposition at 20 μg CDE/mouse i.e., 7.61 µg average CDE/L of chamber air^7^. The aerosol samples were collected immediately after CDE aerosolization using sample collection cassette connected to Gilian 5000 sampler. Chamber air was filtered using the respective air filtration devices for one hour and another chamber air sample was collected after filtration. The analysis of the cat dander Fel d1 levels in all air samples was performed by Indoor Technologies Inc.

Mice were then introduced into the respective chambers for one hour with continuous air filtration during the exposure. Each mouse in the study was observed twice daily for any clinical signs of distress or inactivity. All mice were also weighed weekly during the study period, and finally at necropsy as well. At the end of the study, the lung function parameters were determined for five mice in each group. The rest of the mice in each group were euthanized and blood plasma, BALF, and lung tissue samples were collected and stored for further analysis.

### Lung function analysis

Mice assigned for pulmonary function tests were anesthetized with ketamine/xylazine (K/X) cocktail at a concentration of 80–100/5–10 mg/kg of K/X cocktail, via a single IP injection and the pulmonary functions were tested by forced oscillation techniques using the Buxco® resistance and compliance (R/C) system (Data Sciences International Inc., Holliston, MA). Lung functions were assessed following aerosolized methacholine (MCh) challenges to assess airway hyperresponsiveness (resistance and compliance). Anesthetized mice were intubated via a small superficial incision made in the ventral neck region above the trachea and the trachea was cannulated. Ventilation was performed through the cannula by positive pressure maneuvers on the R/C apparatus. For each mouse, baseline measurements of resistance and compliance were performed that consisted of a slow stepwise or continuous (ramp) inflation to total lung capacity and deflation back to forced respiratory capacity, controlling for pressure. Immediately following baseline measurements, increasing doses of methacholine (in the following order: 0, 1, 3, 6, 12, 25, and 50 mg/mL) were delivered via nebulization and airway resistance (R_L_) and dynamic compliance (C_dyn_) were measured. Mice were then euthanized, and blood plasma and lung tissues were harvested and stored for further analyses.

### Animal necropsy and tissue collection

For the scheduled necropsies, mice were humanely euthanized using Euthasol solution (390 mg sodium pentobarbital/50 mg phenytoin sodium, Virbac AH Inc., Carros, France) solution by intraperitoneal (IP) injection (200 μg/g body wt). Successful euthanasia was confirmed by exsanguination followed by open chest necropsy. Blood plasma was collected in the ethylenediaminetetraacetic acid (EDTA)-coated tubes, and lungs were perfused using PBS solution. The trachea was canulated, the whole lung-heart unit was excised, and BALF was collected using cold PBS. Subsequently, lung tissues were collected and right lung lobes were frozen and left lung lobe was fixed using zinc formalin solution at a constant pressure as described earlier^17^.

### Plasma and BALF cytokine and IgE analysis

Blood plasma was collected in EDTA-coated tubes following centrifugation and aliquots were stored for further analysis. The canulated, excised lungs were lavaged three times with 0.5 mL of cold PBS. For fractionation the collected BALF was centrifuged at 1000×*g* for 10 min at 4 °C and superintend was collected, aliquoted and stored at − 80 °C for further analysis. The remaining cell pellets were re-suspended in 0.2 mL of cold PBS containing 0.1% BSA. From that 20 µL of cell suspension was mixed with equal volume of 0.4% Trypane blue and the total live BAL cells were enumerated by using TC20 automated cell counter (Bio-Rad). Subsequently, 50 µL of cell suspension were cytospun by using Cytospin 2 (Shandon) onto slides for staining procedures. For differential count fixed slides were stained with Camco quik stain II and eosinophils were measured using the BZX700 All-in-one microscopy system (Keyence Inc., Osaka, Japan). Different slide was immunostained for eosinophils (Cat# NBP1-42140; Novus Biologicals, CO) and counter stained with DAPI. Plasma and BALF were analyzed for IgE (Cat# LS-F5589; LSBio, Seattle, WA) and T_H_2 cytokines (e.g., IL-13, Cat# MBS355405; MyBio Source, San Diego, CA) levels by specific ELISAs as per the manufacturers’ instructions.

### Lung tissue histology and immunostaining analysis

The formalin-fixed lungs were then processed for paraffin-embedding and lung tissue sections were prepared. Histochemical staining with hematoxylin and eosin (H&E) was carried out to assess the gross lung morphology. Serial sections were also stained with Alcian Blue (Richard-Allan Scientific) and Periodic acid Schiff’s reagent stain (AB/PAS) to stain for mucin glycoproteins as described earlier^17^. The airway epithelial cell and mucous cell numbers per mm basal lamina^5^ were measured using the BZX700 All-in-one microscopy system (Keyence Inc., Osaka, Japan). Separate sections were immunostained for airway mucin MUC5AC (Cat# MAB2011; Millipore Inc., MA) and eosinophil (Cat# NBP1-42140; Novus Biologicals, CO) following dual-staining technique as described earlier^18^. In all cases, quantification and morphometry was carried out by a person unaware of the slide identity.

### Evaluation protocol for protein degradation on filtration media

The detailed step-by-step Swatch testing protocol was presented in Supplementary Information under Protocol S2.

#### Bovine serum albumin degradation (filter swatch analysis)

A solution of bovine serum albumin (BSA) was used for this initial study at a concentration of 5 mg/mL. Swatches of PECO, carbon, and HEPA filters (1 cm × 1 cm) were used to demonstrate protein degradation. Media was placed under a UV-A light source for no less than 1 h prior to inoculation of protein. 10 µL of the 5 mg/mL solution were pipetted onto swatches under ambient conditions and placed under the UV-A light source for times ranging from 0 to 30 min. Swatches were then placed within 100 µL of 0.5% phosphate buffered solution + Tween-20 (PBST) and vortexed vigorously for one minute. Equal volumes of the solution containing the swatch and 4× Laemmli buffer were transferred to a fresh 1.5 mL flip-cap microcentrifuge tube. Samples were heated to 95 °C for 3–5 min before loading 10 µL into each well of a gel for analysis. The protein ladder was loaded alongside samples according to manufacturer’s guidelines (Thermo Scientific, Spectra Multicolor Broad Range Protein Ladder). Gels were stained according to manufacturer’s guidelines (Thermo Scientific, Gel Code Blue) and de-stained using distilled water. Images of the gel were captured, and the analysis of the images was done by using the open-source Java image processing program, ImageJ. The intensity of the spot on the electrophoresis gel was used to determine the percentage of degradation/adsorption of protein on the respective filter media. Known concentrations of BSA were loaded, run using SDS PAGE and analyzed using ImageJ. The calibration curve (Fig. S6) indicates that analysis via ImageJ can be used reliably and provide accurate information about protein analysis.

#### Aspergillus niger and cat dander protein extractions

Trichloroacetic acid (TCA) precipitation of proteins was performed on crude *Aspergillus niger* and Cat dander extract to reduce the concentration of contaminants and increase the concentration of proteins. 100 µL of TCA were added to 1 mL of crude extract and vortexed vigorously for 30–60 s. Protein precipitated on ice for 30–60 min before centrifugation at 10,000×*g* at 4 °C for 10 min. The supernatant was aspirated, and the pellet was washed two times using 500 µL of ice-cold acetone followed by centrifugation at 10,000×*g* at 4 °C for 5 min. The supernatant was aspirated each time and the pellet was left to air dry. Samples were re-suspended in 50 µL of 0.5% PBST and kept at − 20 °C until further use. Cat dander and *A. niger* protein extracts were subjected to similar treatment of BSA degradation using swatches of PECO-coated (media (1 cm × 1 cm). Images were captured of the gel followed by analysis using the open-source Java image processing program, ImageJ.

### Proteomic analysis

Next, we performed proteomic analysis of CDE for that equal volumes of protein (100 ul) extracted from swatches of filter media were processed for LC–MS/MS using s-traps (Protifi) (Fig. S11)^31,32^. Proteins were reduced with dithiothreitol (DTT), alkylated with iodoacetamide (IAA), acidified using phosphoric acid, and combined with s-trap loading buffer (90% methanol, 100 mM TEAB). Proteins were loaded onto s-traps, washed, and finally digested with Trypsin/Lys-C overnight at 37 °C. Peptides were eluted and dried with a vacuum concentrator. Peptides were resuspended in H_2_O/1% acetonitrile/0.1% formic acid for LC–MS/MS analysis.

Peptides were separated using a 75 µm × 50 cm C18 reversed-phase-HPLC column (Thermo Scientific) on an Ultimate 3000 UHPLC (Thermo Scientific) with a 120-min gradient (2–32% ACN with 0.1% formic acid) and analyzed on a hybrid quadrupole-Orbitrap instrument (Q Exactive Plus, Thermo Fisher Scientific). Full MS survey scans were acquired at 70,000 resolutions. The top 10 most abundant ions were selected for MS/MS analysis.

Raw data files are processed in MaxQuant (v 2.1.4, www.maxquant.org) and searched against the current Uniprot *Felis catus* protein sequences database. Search parameters included constant modification of cysteine by carbamidomethylation and the variable modifications, methionine oxidation and protein N-term acetylation. Proteins were identified using the filtering criteria of 1% protein and peptide false discovery rate. Protein intensity values were normalized using the MaxQuant-label free quantitation (LFQ) function^33^.

### Statistical analysis

Grouped results were expressed as means ± SEM and p < 0.05 was considered significant. Data were analyzed using GraphPad Prism Software (GraphPad Software Inc., San Diego, CA) using a one-way analysis of variance (ANOVA) or a two-way ANOVA and with a post-hoc multiple comparison analysis. When significant effects were detected (p < 0.05), Fisher’s least significant difference and student’s t test was used to determine differences between groups.

### Ethics declaration

All methods were carried out in accordance with relevant guidelines and regulations. All methods are reported in accordance with ARRIVE guidelines (https://arriveguidelines.org).

## Supplementary Information

Supplementary Information.

## Data Availability

All data generated or analyzed during this study are included in this published article and its Supplementary Information files.
