# Supplementary material for: Indoor-air purification by photoelectrochemical oxidation mitigates allergic airway responses to aerosolized cat dander in a murine model
Source: Sci Rep. 2023 Jul 6;13:10980. doi: 10.1038/s41598-023-38155-0 (PMC10325967; doi:10.1038/s41598-023-38155-0)
Supplement: Supplementary file 1 — Supplementary Information. [file 41598_2023_38155_MOESM1_ESM.docx]

**Supporting Information**

**Indoor-Air Purification by Photoelectrochemical Oxidation Mitigates Allergic Airway Responses to Aerosolized Cat Dander in a Murine Model**

*Dinesh Devadoss^1,^***, Kerri Surbaugh^2^, Marko Manevski^1^, Chatura Wickramaratne^2^, Dale Chaput^3^, Arianne Chung^1^, Francisco de Leon^1^, Hitendra S. Chand^1^, and Jaspreet S. Dhau^2,^**

^1^Department of Immunology and Nano-Medicine, Herbert Wertheim College of Medicine, Florida International University, 11200 SW 8^th^ St, Miami, FL 33199, USA

^2^Molekule, Inc., 3802 Spectrum Blvd, Tampa, FL 33612, USA

^3^ Department of Microbiology, University of South Florida, Tampa, FL-33612

***Corresponding Authors:** Jaspreet S. Dhau, PhD

Molekule, Inc.,

3802 Spectrum Blvd,

Tampa, FL 33612

E-mail: [jasdhau@gmail.com](mailto:jasdhau@gmail.com); [jdhau@molekule.com](mailto:jdhau@molekule.com)

Dinesh Devadoss, PhD

Department of Immunology and Nano-Medicine,

Herbert Wertheim College of Medicine,

Florida International University,

Miami, FL 33199

E-mail: [ddevados@fiu.edu](mailto:ddevados@fiu.edu)

**Running title:** Indoor-air Filter Mitigates Allergic Asthma in Murine Model

**
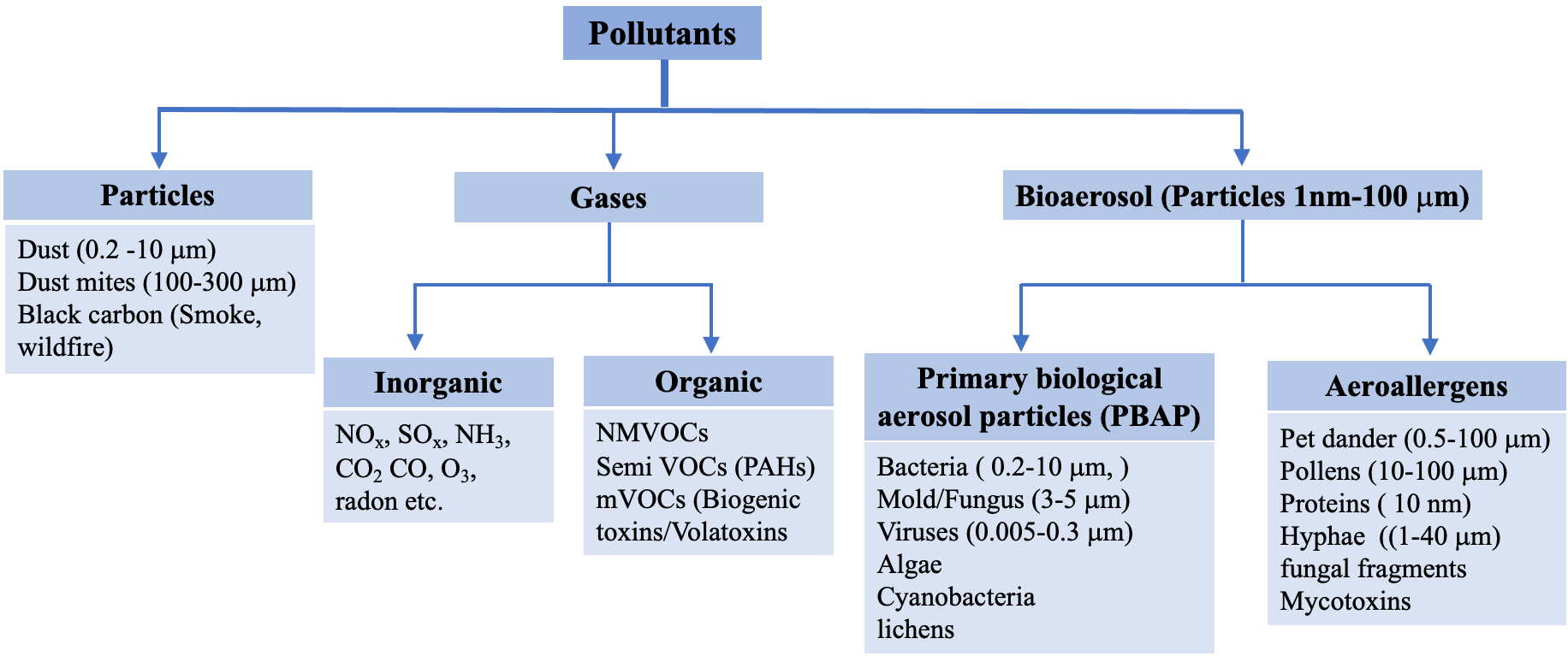
**

**Fig. S1.** Composition of indoor air pollutants. **Biogenic toxins**: alcohols, aldehydes, ketones, amines, terpenes, aromatics, halogenated and sulfur-based compounds. **NMVOCs:** non-methane volatile organic compounds, **PAH:** Polyaromatic hydrocarbons.


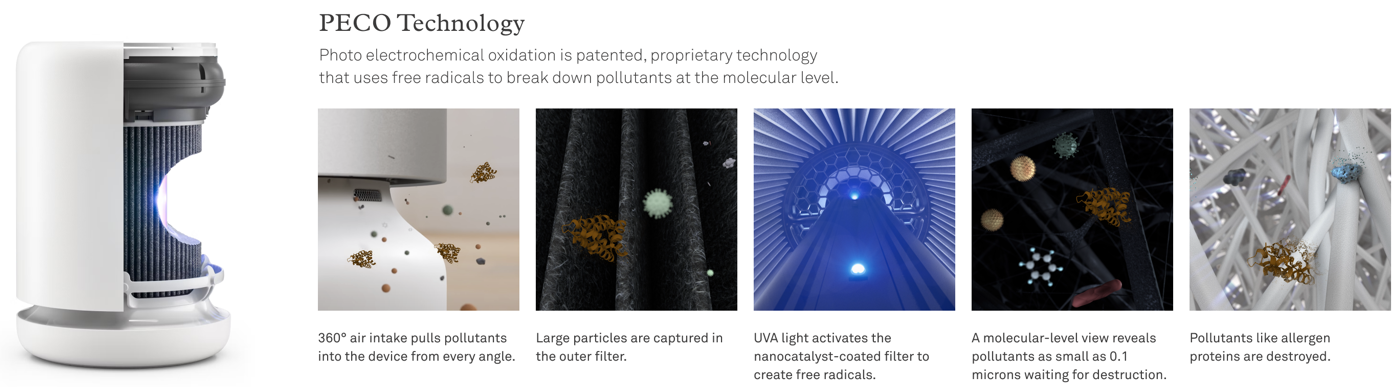

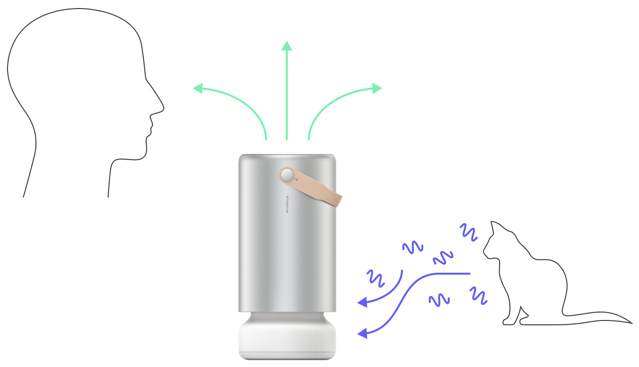

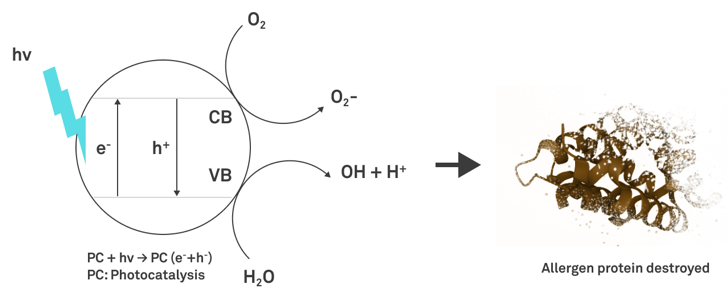


**Fig. S2.** Schematic representation of photoelectrochemical oxidation-assisted air purification and interaction with allergen proteins.

**Protocol S1: Cat Dander Extract Aerosolization, Air Sampling and Exposure Procedure**

Four test-chambers (**Fig. S3**) of ~4000L capacity were used for the study. Three of these chambers were exposed to Cat Dander Extract (CDE). Each of the three allergen-challenged chambers had one of the following:

1. Air Mini + with Photoelectrochemical oxidation (PECO) assisted purifier
2. HEPA-filter utilizing air purifier
3.
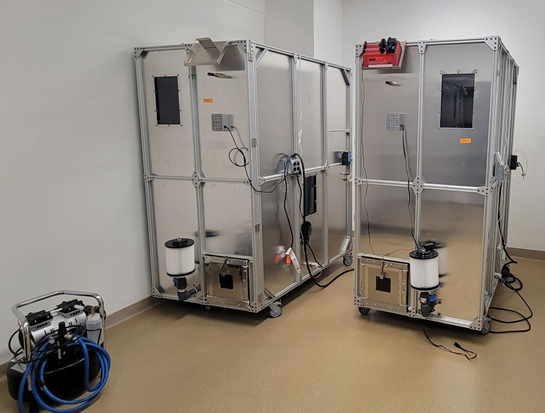
Air Mini + (Sham filter) as a Positive control

**Fig. S3.** The custom-built whole-body aerosol exposure chambers were used in the study.

One chamber was not exposed to cat dander and served as a negative control. This chamber had an Air Mini + device. Crude cat dander extract was nebulized into the exposure chambers. A starting concentration air sample was taken directly after nebulization. Then, the air cleaner was run for 1.0 hour. At the end of the 1 hour, an additional air sample was taken. While the air cleaners continue to run, mice (n=10) were introduced to the chambers. Mice remained in the chambers for 1.0 hour while the air cleaning device continued to run. Mice were then removed from the chambers and the air cleaning devices were turned off. These exposure events took place three times a week for six weeks, making a total of 18 exposure events per chamber. After
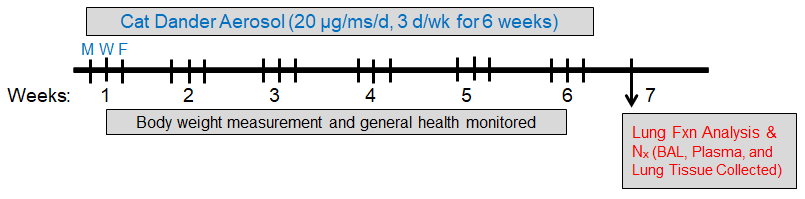
these events, mice were necropsied, and biomarkers observed.

**Fig. S4.** The schematics of the study design followed for the experimental animal model of CDE-induced allergic asthma.

**Equipment and Consumables**

**Table S1** lists all equipment and consumables used in the study.

| Test chambers (4) | Molekule Air Minis +  True HEPA-filter Device | Molekule Air Mini Filter (1) |
| --- | --- | --- |
| 1/16” ID Tygon tubing (BLAM feed line) with Luer lock nuts | Blaustein Atomizing Module (BLAM) – 8 Jet | Syringe pump and BD syringes |
| Air compressor and air hose with quick connect | Gilian 5000 sampling pump (2) | 37 mm air sampling cassettes loaded with fiberglass filters (150) |
| 1/4” Tygon tubing with cassette fittings | Sampler port caps | Hygro/thermometers (4) |
| 1-5LPM Chek-mate calibrator | 1.08 L PBS, 7.4 pH 1X | 216 mL Cat dander extract (54 cryotubes) |
| Mice (40) and cages | 1” wrenches (2) | Nebulizer plugs (4) |
| HEPA outlet filters (8) | Programmable, NIST traceable timers (4) |  |

**Cat Dander Extract Dose Calculation**

The target dose for each mouse was set at 20 mg of cat dander extract (CDE) during the 1 h in duration exposures, if the tidal volume of mouse breath per minute is 1.46 mL air/gram of mouse mass ([Milton et al. 2012](https://drive.google.com/file/d/1NHIpoiztKTqkhc7lWCWppErqW45Y81_f/view?usp=sharing))([Fairchild 1972](https://drive.google.com/file/d/1_SK2TG8AvJGjRi-7WZUgVQAqrP2oBbrJ/view?usp=sharing)), then a 30 g mouse should breathe an average of 2.628 L of air over 60 min. In order to get 20 mg of cat dander delivered to that mouse via inhalation in that 2.628 L of air, the air concentration in the chamber should be **7.61** mg CDE/L of air. Therefore, 30.44 mg CDE is needed for each chamber (each chamber is approx. 4000 L).


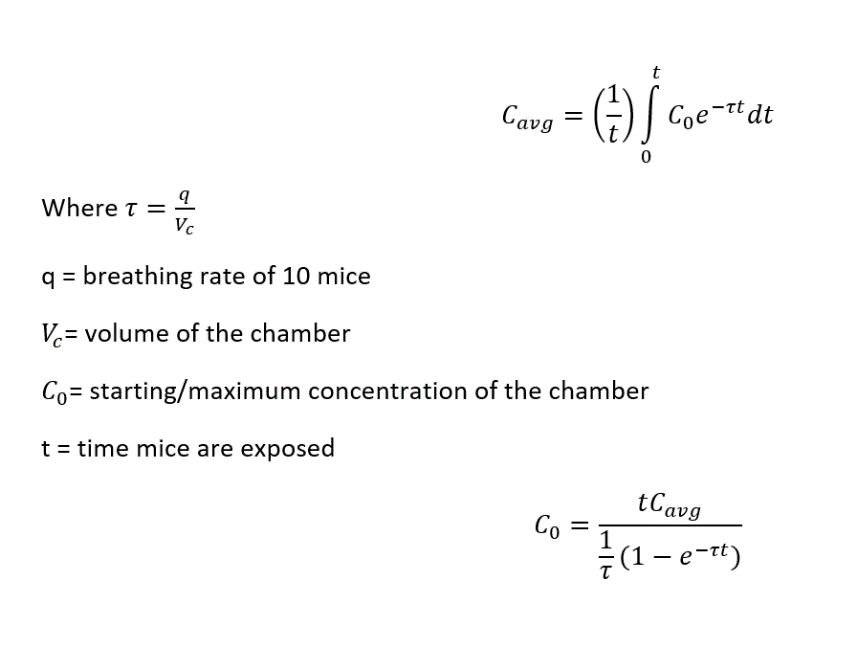


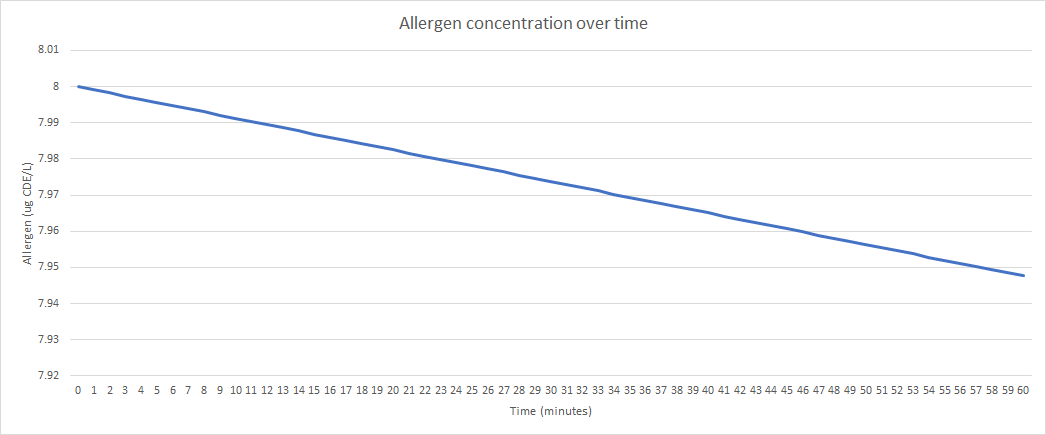


**Fig. S5.** Natural decay of CDE in the chamber during the 1 h duration.

Considering the natural decay (30%) of CDE in the chamber during the 60-minute duration (Fig. S5) and 18 days of exposure in three chambers, the amount of CDE needed for the study was determined to be 2.14 kg.

**Prep the Air Mini + Air Cleaning Devices:**

1. Place an Air Mini + unit in the PECO testing chamber. Choose one of the brand-new production filters. Inspect the filter and verify that no damage occurred in shipping. If the filter looks good, proceed with the use of this filter. Unwrap the filter and place the filter in the Air Mini + device.
2. Plug in the Air Mini + air cleaner into the provided extension cord in the PECO testing chamber. Plug the extension cord into the electrical box on the outside of the chamber. Use the iPhone provided (or press the center button manually) to set the Air Mini + to the top fan setting (**Speed 5**). The fan speed is indicated by the number of white LEDs lit on the lid—i.e., the lid will display five (5) white LEDs when running at Speed 5. Unplug the extension cord from the electrical box.
3. Repeat steps 1&2 for the Air Mini + device located in the Negative Control chamber.
4. Set an Air Mini + device in the main testing room, near to the door that connects the main testing area with the Negative Control testing and animal housing room. Select a filter, document the filter serial number, and load the filter into the Air Mini +. This air cleaner is meant to help prevent contamination reaching the animal housing area. Set this air cleaner at the highest level that is comfortable for the technicians. Leave this unit running perpetually.
5. Repeat step 1 for the “clean up” Air Mini + unit in the Positive Control chamber *(the device that will ONLY be used to clean up the chamber* ***after the exposure for the mice has concluded****)*. For this device, plug the Air Mini + into the extension cord provided in the chamber, **but make sure the device is turned off.***
6. The Positive Control chamber will have a placebo air cleaner in addition to the “clean up” air cleaner. The placebo air cleaner in the Positive Control chamber should **NOT** have a filter in it. Its firmware will have been updated to run without a filter or internal lights.
7. Place this special, placebo filter in the center of the chamber (off-set slightly to permit room for the mice cages), plug into the extension cord, and verify that the unit starts.
8. Keep both air cleaners plugged into the extension cord and the extension cord plugged into the outlet box. Keep both air cleaners off until needed. Use the iPhone or the chamber glove to start either the placebo or the “clean up” Air Mini device when these units are needed.

** It is critical that this device does not accidentally clean the air of the Positive Control chamber. The chamber glove will be used to turn this device on manually when it is needed (post-experiment).*

**Prep the HEPA Filtered Air Cleaning Device:**

1. Verify that the HEPA filtered device is plugged into the extension cord in the HEPA chamber. Plug the extension cord into the external electrical box on the chamber. Press the power button to turn the device on. The device should be set to the same fan speed as the Air Mini+ device. *Note: the HEPA-filter utilizing air cleaner will not automatically turn on when plugged in. During the experiment, the glove box will need to be used to press the power button to start this air cleaner.*

**Prep the Chambers for Testing Day:**

1. Check the latches that secure the air-tight seal on the doors. The latches have threading that allows them to be tightened. The latches should emit a loud snap when closed. Reminder, cat dander leaking from chambers could lead to cross contamination and invalidation of the study. **Be sure that all 6 latches on each chamber create a tight seal.**
2. Open all chambers.  Spray 70% ethanol onto all surfaces of the chamber. Use the T-mop provided (or paper towels) to wipe down the inside of the chambers. Leave the doors open while the chambers evaporate the excess ethanol. Make sure that the testing room Air Mini + is running to help absorb these fumes.
3. Verify that all sampling ports are sealed with a rubber cap.
4. Verify that all nebulizing ports are sealed with the provided steel plug.
5. Plug the mixing fan cord into the outlet box on the outside of each chamber. This fan will run throughout the entire experiment.
6. Verify that the extension cord is NOT plugged into the outlet box on each chamber--with the exception of the Positive Control chamber. The Positive Control chamber requires the extension cord to be plugged in, but both Air Minis within to be turned off.
7. Verify that the nebulizing port relief valve has a HEPA filter secured to the outlet. Open the nebulizing relief port valve slightly. Change these HEPA filters on Week 3.
8. Verify that the air cleaners are in position—centered, off-set to the far end of the chamber so that the cages of mice can easily fit in a central location as well. For the positive control chamber, verify that the clean-up Mini is set in the corner of the chamber (out of the way), next to the nebulizing relief valve outlet.

**Prep the Sampling Pump and Cassettes for Testing Day:**

1. Label eight (8) 37 mm air sampling cassettes using general lab or cryo-tape with the following:
   1. Date
   2. Chamber
   3. Sample number (1 or 2)
2. Calibrate the sampling pumps weekly. Use a fresh 37 mm air sampling cassette each week. Label the cassettes for easy identification. 150 air sampling cassettes were provided. This means that there are 144 for experimental samples and 6 cassettes for calibration.
3. To calibrate the pumps, follow these steps:

Connect a fresh sampling cassette to the Chek-mate calibrator. Verify that the short tube fitting is the connection between the calibrator and the “Inlet” opening of the 37 mm cassette. Connect the longer tube from the “Outlet” opening of the 37 mm cassette to the Gilian 5000 sampling/vacuum pump.

- 1. Turn on the Chek-mate calibrator.
  2. Turn on the Gilian 5000 sampling pump.
  3. Press the “Set/Cal” button twice so that “CAL” is displayed on the screen.
  4. Press the “Enter” button to enter calibration mode.
  5. When the pump starts, the flow rate setting will be displayed on the screen of the Gilian 5000. The true sampling flow rate will be displayed on the Chek-mate calibrator at the same time.
  6. Use the up and down buttons on the Gilian 5000 to adjust the flow rate displayed on the Gilian 5000 so that it matches the flow rate displayed on the Chek-mate as closely as possible.
  7. Press “Enter” to complete calibration.
  8. If the difference between the flow rate shown on the Gilian 5000 and the Check-mate is greater than 1%, repeat items (f) through (h). If the difference is less than 1%, calibration is complete.

1. Fill out the Indoor Biotechnologies sample form to reflect the eight samples of the day

**Prep the Aerosol Equipment:**

1. Place the syringe pump in the pump holder on the testing chamber. Plug the pump into the outlet box on the chamber.
2. Use the switch on the upper left of the pump to turn the pump on.
3. Press “Rate” to verify the pumping rate is set to **0.4 mL/min**.
4. Press “Diameter” to verify that the ID shown on the syringe pump display matches the ID of the syringe used. Use the back page of the [manual](about:blank) to check internal dimension values. For example, if a 10mL syringe is used, the ID setting on the syringe pump should be 14.43mm.
5. Draw 4 ml of cat dander extract (CDE) into a sterile syringe.
6. Fasten the Luer lock nut connector with 1/16” ID Tygon tubing onto the tip of the syringe.
7. Lift the syringe clamp on the syringe pump and set the syringe in the groove of the pump. Verify that the plunger flange is set in the plunger flange groove by the anti-siphon plate. Verify that the barrel flanges are set in the syringe retainer bracket. *Note: If both the barrel flanges and the plunger flange are not secured, the immense vacuum pressure of the Blaustein Atomizer will empty the syringe immediately.* ***Practice the use of the syringe pump prior to use with cat dander.***
8. If any CDE has been injected into the Tygon tubing (prematurely), use the drive-nut button to gently shift the pusher block to the left to withdraw the solution back into the syringe.
9. Press the “Start” button. Verify that a steady light appears by “Pumping”. Press “Start” again to pause the pumping. The light next to “Pumping” should be blinking.
10. Make sure that the liquid feed line (1/16” Tygon tubing with the Luer lock fitting) connects both to the syringe as well as the liquid feed port of the BLAM. Trim any unnecessary line from connection between the liquid feed port on the BLAM and the Luer fitting of the syringe*. If this line is too long, CDE will hang in the line and not nebulize.*
11. Verify that the liquid feed tube within the BLAM connects both to the liquid feed port as well as the atomization nozzle.
12. Fill the BLAM jar with 15 mL of 7.4 pH sterile PBS.
13. Fasten the BLAM to the chamber using the Swagelok fittings on the nebulizing port. Tighten using the set of wrenches (1” and 1-1/16”) provided.
14. Plug in the P120-24 air compressor. Verify that the air compressor is positioned close enough to the chamber for the pneumatic line with quick connect to reach the BLAM. *Do not connect the pneumatic line to the BLAM until it is time to nebulize into the chamber.*
15. Check the tank drain jar. Empty any accumulated water.
16. Set the switch on the compressor to “Auto”.
17. **Set the air compressor to 40 psig** using the regulator and air gun. The compressor needs to be pumping air to regulate the flow.

**Exposure Protocol for the PECO chamber:**

1. Print the checklist/protocol for each chamber and keep next to the chamber. Use of a clipboard is recommended. Document the date:
   1. Date: _________________________
2. Shut the chamber door. **Make sure that all six of the door clamps are tightly fastened.**
3. Connect the ¼” Tygon tubing connection to the sampling port of the chamber.  Trim any excess Tygon so that the line from the chamber sampling port to the sampling cassette is as short as possible—*without creating a sharp bend in the line*. The shorter the line, the less CDE will get trapped before reaching the cassette. Gradual curves are best.
4. Verify that the Luer fitting of the Tygon line is connected to the “Inlet” side of the sampling cassette. Verify that a Luer fitting with Tygon line is connected securely to both the “Outlet” side of the cassette and the Gilian 5000 sampler. Turn on the sampler pump so that the resting screen rotation is displayed.
5. Check the nebulizing relief port to ensure that the valve is opened slightly (to reduce pressure buildup from nebulizing).
6. Clear the memory of the Durac hygrometer. Document the starting temperature and relative humidity:
   1. Temperature: _________________________
   2. Relative Humidity: _________________________
7. Connect the pneumatic line to the BLAM. Press “Start” on the syringe pump to begin pumping CDE into the exposure chamber. Document the time.
   1. Time: _________________________
8. Set the timer on the chamber for 10 minutes. Permit the BLAM to nebulize CDE into the chamber until the timer goes off. Take a moment to watch the fluid in the liquid feed line from the syringe to the BLAM. Movement toward the BLAM should be visibly evident.
9. Check the syringe. Verify that all the CDE has been nebulized into the chamber. Allow the BLAM to nebulize until all CDE has been injected.
10. Shut the nebulizer relief valve.
11. Take the first sample by pressing and holding the “Run/Stop” button until “SCAL” shows on the screen. Listen for the pump whirring sound to verify that the pump has started. The pump should automatically stop after 2 minutes.
12. Remove the cassette from the fittings. Use a rubber cap to seal the sampling port until the next use.
13. Store the used 37 mm air sample cassette in the refrigerator or on ice until it can be shipped to Indoor Biotechnologies.
14. Loosen the Swagelok fittings that hold the BLAM onto the chamber. Remove the BLAM and use the steel plug to close the nebulizing port. Tighten using a wrench.
15. Plug in the extension cord into the outlet box on the outside of the chamber. Watch the Air Mini + start up. **Verify that all 5 dots of light are visible on the top of the Air Mini +, indicating that the air cleaner is set to the highest fan speed.**
16. Note the time:
    1. Time: _________________________
17. Start the 1-hour timer. Permit the Air Mini + to run for 1 hour.
18. Take the second air sample using the Gilian 5000 and the fresh 37 mm cassette.
19. Introduce the cages of mice into the chamber, using the pass-through box and the chamber glove.
20. Note the time:
    1. Time: _________________________
21. Start the 1-hour timer.
22. Remove the mice from the chamber after the 1 hour of exposure has concluded.
23. Document the min. and max. temperature and relative humidity for the duration of the experiment as shown on the Durac hygrometer:
24. Min and Max Temperature: _________________________
    1. Min and Max Relative Humidity: _________________________
25. Proceed to the clean-up protocol.

**Exposure Protocol for the HEPA Chamber:**

1. Print the checklist/protocol for each chamber and keep next to the chamber. Use of a clipboard is recommended. Document the date:
2. Date: _________________________
3. Shut the chamber door. **Make sure that all six of the door clamps are tightly fastened.**
4. Connect the ¼” Tygon tubing connection to the sampling port of the chamber.  Trim any excess Tygon so that the line from the chamber sampling port to the sampling cassette is as short as possible—*without creating a sharp bend in the line.* The shorter the line, the less CDE will get trapped before reaching the cassette. Gradual curves are best.
5. Verify that the Luer fitting of the Tygon line is connected to the “Inlet” side of the sampling cassette. Verify that a Luer fitting with Tygon line is connected securely to both the “Outlet” side of the cassette and the Gilian 5000 sampler. Turn on the sampler pump so that the resting screen rotation is displayed.
6. Check the nebulizing relief port to ensure that the valve is opened slightly (to reduce pressure buildup from nebulizing).
7. Clear the memory of the Durac hygrometer. Document the starting temperature and relative humidity:
   1. Temperature: _________________________
   2. Relative Humidity: _________________________
8. Connect the pneumatic line to the BLAM. Press “Start” on the syringe pump to begin pumping CDE into the exposure chamber. Document the time.
   1. Time: _________________________
9. Set the timer on the chamber for 10 minutes. Permit the BLAM to nebulize CDE into the chamber until the timer goes off. Take a moment to watch the fluid in the liquid feed line from the syringe to the BLAM. Movement toward the BLAM should be visibly evident.
10. Check the syringe. Verify that all the CDE has been nebulized into the chamber. Allow the BLAM to nebulize until all CDE has been injected.
11. Shut the nebulizer relief valve.
12. Take the first sample by pressing and holding the “Run/Stop” button until “SCAL” shows on the screen. Listen for the pump whirring sound to verify that the pump has started. The pump should automatically stop after 2 minutes.
13. Remove the cassette from the fittings. Use a rubber cap to seal the sampling port until the next use.
14. Store the used 37 mm air sample cassette in the refrigerator or on ice until it can be shipped to Indoor Biotechnologies.
15. Loosen the Swagelok fittings that hold the BLAM onto the chamber. Remove the BLAM and use the steel plug to close the nebulizing port. Tighten using a wrench.
16. Plug in the extension cord into the outlet box on the outside of the chamber. Use the glove to press the power button on the top of the HEPA-filter utilizing air cleaner. The HEPA-filter utilizing will light up as it turns on.
17. Note the time:
18. Time: _________________________
19. Start the 1-hour timer. Permit the HEPA-filter utilizing air purifier to run for 1 hour.
20. Take the second air sample using the Gilian 5000 and the fresh 37 mm cassette.
21. Introduce the cages of mice into the chamber, using the pass-through box and the chamber glove.
22. Note the time:
    1. Time: _________________________
23. Start the 1-hour timer.
24. **Remove the mice from the chamber after the 1 hour of exposure** has concluded.
25. Document the min. and max. temperature and relative humidity for the duration of the experiment as shown on the Durac hygrometer:
26. Min and Max Temperature: _________________________
    1. Min and Max Relative Humidity: _________________________
27. Proceed to the clean-up protocol.

**Exposure Protocol for the Positive Control chamber:**

1. Print the checklist/protocol for each chamber and keep next to the chamber. Use of a clipboard is recommended. Document the date:
2. Date: _________________________
3. Shut the chamber door. **Make sure that all six of the door clamps are tightly fastened.**
4. Connect the ¼” Tygon tubing connection to the sampling port of the chamber.  Trim any excess Tygon so that the line from the chamber sampling port to the sampling cassette is as short as possible—*without creating a sharp bend in the line.* The shorter the line, the less CDE will get trapped before reaching the cassette. Gradual curves are best.
5. Verify that the Luer fitting of the Tygon line is connected to the “Inlet” side of the sampling cassette. Verify that a Luer fitting with Tygon line is connected securely to both the “Outlet” side of the cassette and the Gilian 5000 sampler. Turn on the sampler pump so that the resting screen rotation is displayed.
6. Check the nebulizing relief port to ensure that the valve is opened slightly (to reduce pressure buildup from nebulizing).
7. Clear the memory of the Durac hygrometer. Document the starting temperature and relative humidity:
   1. Temperature: _________________________
   2. Relative Humidity: _________________________
8. Connect the pneumatic line to the BLAM. Press “Start” on the syringe pump to begin pumping CDE into the exposure chamber. Document the time.
   1. Time: _________________________
9. Set the timer on the chamber for 10 minutes. Permit the BLAM to nebulize CDE into the chamber until the timer goes off. Take a moment to watch the fluid in the liquid feed line from the syringe to the BLAM. Movement toward the BLAM should be visibly evident.
10. Check the syringe. Verify that all the CDE has been nebulized into the chamber. Allow the BLAM to nebulize until all CDE has been injected.
11. Shut the nebulizer relief valve.
12. Take the first sample by pressing and holding the “Run/Stop” button until “SCAL” shows on the screen. Listen for the pump whirring sound to verify that the pump has started. The pump should automatically stop after 2 minutes.
13. Remove the cassette from the fittings. Use a rubber cap to seal the sampling port until the next use.
14. Store the used 37 mm air sample cassette in the refrigerator or on ice until it can be shipped to Indoor Biotechnologies.
15. Loosen the Swagelok fittings that hold the BLAM onto the chamber. Remove the BLAM and use the steel plug to close the nebulizing port. Tighten using a wrench.
16. Start the placebo Air Mini. Verify that the “clean up”, filtered Air Mini + in the corner of the chamber has **NOT** started (i.e., remains **OFF**).
17. Note the time:
18. Time: _________________________
19. Start the 1-hour timer. Permit the placebo Air Mini + (with no filter and no lights) to run for 1 hour.
20. Take the second air sample using the Gilian 5000 and the fresh 37 mm cassette.
21. Introduce the cages of mice into the chamber, using the pass-through box and the chamber glove.
22. Note the time:
    1. Time: _________________________
23. Start the 1-hour timer.
24. **Remove the mice from the chamber after the 1 hour of exposure** has concluded.
25. Document the min. and max. temperature and relative humidity for the duration of the experiment as shown on the Durac hygrometer:
26. Min and Max Temperature: _________________________
    1. Min and Max Relative Humidity: _________________________
27. Start the “clean up” Air Mini + unit running to clean the chamber prior to opening. Permit the Air Mini + to run at least 15 minutes (a longer period of time is better).
28. Proceed to the clean-up protocol.

**Exposure Protocol for the Negative Control chamber:**

1. Print the checklist/protocol for each chamber and keep next to the chamber. Use of a clipboard is recommended. Document the date:
2. Date: _________________________
3. Shut the chamber door. **Make sure that all six of the door clamps are tightly fastened.**
4. Connect the ¼” Tygon tubing connection to the sampling port of the chamber.  Trim any excess Tygon so that the line from the chamber sampling port to the sampling cassette is as short as possible—*without creating a sharp bend in the line.* The shorter the line, the less CDE will get trapped before reaching the cassette. Gradual curves are best.
5. Verify that the Luer fitting of the Tygon line is connected to the “Inlet” side of the sampling cassette. Verify that a Luer fitting with Tygon line is connected securely to both the “Outlet” side of the cassette and the Gilian 5000 sampler. Turn on the sampler pump so that the resting screen rotation is displayed.
6. Clear the memory of the Durac hygrometer. Document the starting temperature and relative humidity:
   1. Temperature: _________________________
   2. Relative Humidity: _________________________
7. Take [the first sample](https://youtu.be/PkbBGSuLz7M) by pressing and holding the “Run/Stop” button until “SCAL” shows on the screen. Listen for the pump whirring sound to verify that the pump has started. The pump should automatically stop after 2 minutes.
8. Remove the cassette from the fittings. Use a rubber cap to seal the sampling port until the next use.
9. Store the used 37 mm air sample cassette in the refrigerator or on ice until it can be shipped to Indoor Biotechnologies.
10. Loosen the Swagelok fittings that hold the BLAM onto the chamber. Remove the BLAM and use the steel plug to close the nebulizing port. Tighten using a wrench.
11. Plug in the extension cord into the outlet box on the outside of the chamber. Watch the Air Mini + start up. **Verify that all 5 dots of light are visible on the top of the Air Mini +, indicating that the air cleaner is set to the highest fan speed.**
12. Note the time:
13. Time: _________________________
14. Start the 1-hour timer. Permit the Air Mini + to run for 1 hour.
15. Take the second air sample using the Gilian 5000 and the fresh 37 mm cassette.
16. Introduce the cages of mice into the chamber, using the pass-through box and the chamber glove.
17. Note the time:
    1. Time: _________________________
18. Start the 1-hour timer.
19. **Remove the mice from the chamber after the 1 hour of exposure** has concluded.
20. Document the min. and max. temperature and relative humidity for the duration of the experiment as shown on the Durac hygrometer:
21. Min and Max Temperature: _________________________
    1. Min and Max Relative Humidity: _________________________
22. Start the “clean up” Air Mini + unit running to clean the chamber prior to opening. Permit the Air Mini + to run at least 15 minutes (a longer period of time is better).
23. Proceed to the clean-up protocol.

**Clean-up Protocol:**

1. Refrigerate air samples until they can be shipped to Indoor Biotechnologies. Fill out the sample form to reflect the samples included with the shipment. Place this form in a plastic bag with the samples.
2. Ship the samples on regular or dry ice, FedEx standard overnight to the address listed on the form. Ship samples every Tuesday for the duration of the exposures.
3. Return mice to cage racking.
4. Open each chamber and spray the interior with 70% ethanol. Use paper towels or cloths to wipe any deposited CDE from the walls and floor of the chamber.
5. Leave the chamber doors open to air out.
6. Dispose of the used syringe.
7. Wash the BLAM with laboratory glass cleaner, rinse well with tap and distilled water.
   1. **Do not use bleach on the BLAM.** The corrosive nature of hypochlorite is damaging. If sterilization is desired, the BLAM can be soaked in 70% ethanol or autoclaved (after all plastic and o-rings have been removed).
8. Rinse Tygon tubing and fittings with 70% ethanol and allow to completely dry. **Only the tubing and fittings that sit between the chamber and the cassette need cleaning.** The tubing that runs from the cassette to the vacuum pump is not necessary to clean. Similarly, **use of bleach is not recommended.**

**Protocol S2: Swatch Testing Protocol for Protein Degradation Efficiency**

The swatch testing was done to determine the protein degradation efficiency of various filtration media. By using a swatch of media and a known concentration of protein, degradation can be determined using a SDS PAGE (**P**oly**A**crylamide **G**el **E**lectrophoresis). The procedure followed for the analysis is given below.

1. Apply 10 microliters of the 5 mg/mL concentration of protein to each of the swatches of media.
2. Place media under UV-A light for desired time (0-60 minutes).
3. Using DRY, 10% bleach sterilized forceps remove swatch of media from UV-A light and place within a 1.5 mL flip-cap microcentrifuge tube containing 100 microliters of 0.5% Tween 20 H_2_O and push to the bottom of the tube without immersing the forceps.
4. Vortex 1.5 mL flip-cap microcentrifuge tube containing 100 microliters of 0.5% Tween 20 H_2_O and the swatch of media for 30-60 seconds.
5. Centrifuge 1.5 mL flip-cap microcentrifuge tube containing 100 microliters of 0.5% Tween 20 H_2_O and the swatch of media for 30-60 seconds.
6. Repeat Steps **5 and 6**.
7. Grasp swatch of media by a corner and pull to the top of the 1.5 mL flip-cap microcentrifuge tube and close the flip-cap onto the corner of the swatch of media. This step is important for removing drying the swatch of media and obtaining a usable sample.
8. Centrifuge the sample two times for 30-60 seconds each time, allowing the centrifuge to completely stop before beginning the second run.

**Note: There should be between 80-100 microliters of sample. If there is less than 80 microliters, some of your sample is probably still within the swatch and further centrifugation is required.** **Do not perform step 10 unless there is enough volume.**

1. Carefully open the 1.5 mL flip-cap microcentrifuge tube and remove the swatch of media.
2. Pipette equal volumes of your sample and loading buffer into a clean 1.5 mL flip-cap microcentrifuge tube (i.e., 15 microliters of sample and 15 microliters of loading buffer).
3. Put tube containing loading buffer and sample into a 95˚C heat block for 3-5 minutes.
4. Run sample on a 4-20% gradient gel with fifteen, 15 microliter, wells at 150V for ~30-50 minutes, or until desired separation is achieved.
5. Wash the gel with double distilled H_2_O with gentle rocking for 5 minutes.
6. Repeat step 14 two more times.
7. Add ~20 mL of Gel Code Blue to the gel and agitate with gentle rocking for at least one hour. For best results, cover with aluminum foil (or Parafilm) and keep overnight.
8. Wash the gel with double distilled H_2_O with gentle rocking for 5 minutes to de-stain the Gel Code Blue.
9. Repeat step 17 two more times.
10. Take a picture of the gel for records and further analysis.

**Calibration Curve**

Known concentrations of BSA were loaded and run using SDS PAGE and analyzed using imageJ. This calibration curve [Fig. S6] indicates that analysis via imageJ can be used reliably and provide accurate information about protein degradation analysis. Electrophoresis gel images are displayed in Figs. S7-S11.


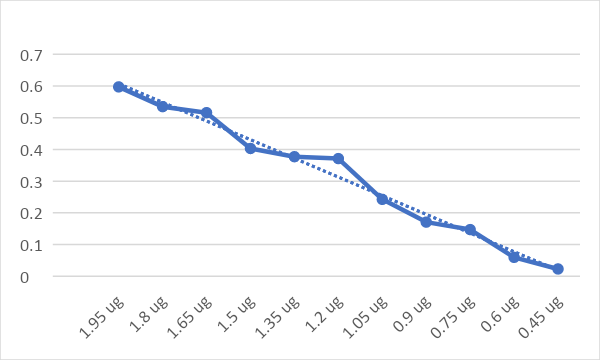


**Fig. S6.** Calibration curve with BSA assay (SDS PAGE)**.**


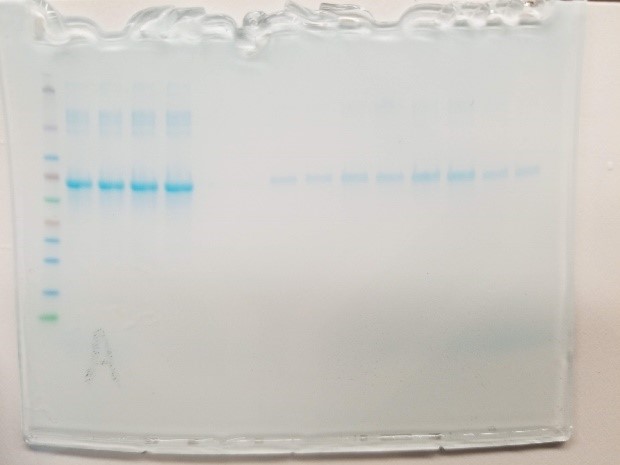


BSA HEPA PECO 2.0 PECO 1.0 Carbon

**Fig. S7.** Electrophoresis gel image of *BSA* degradation assay


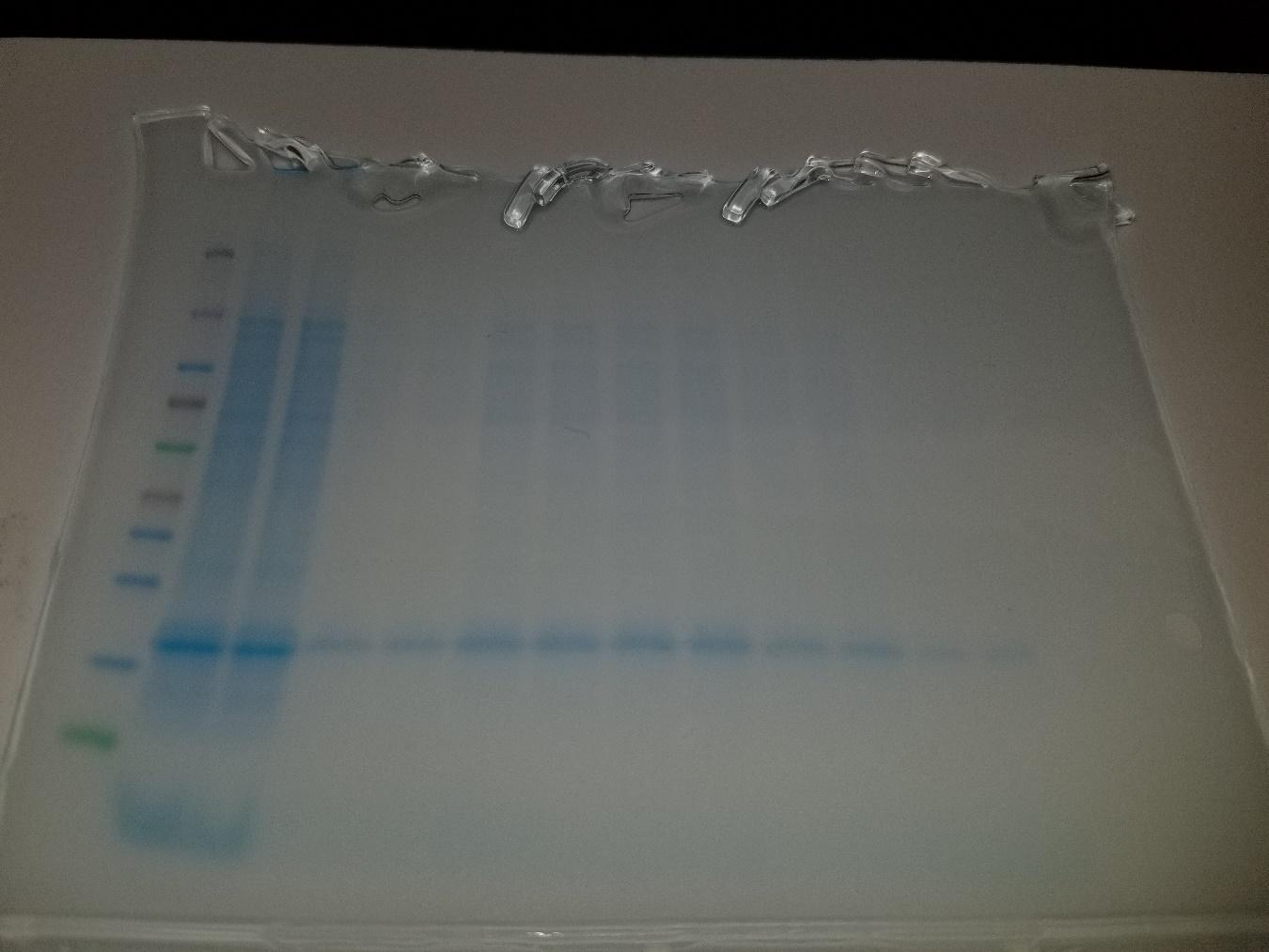


1. Niger protein extract

Control

PECO

**Fig. S8.** Electrophoresis gel image of *A. niger* protein extract degradation assay (5 min).


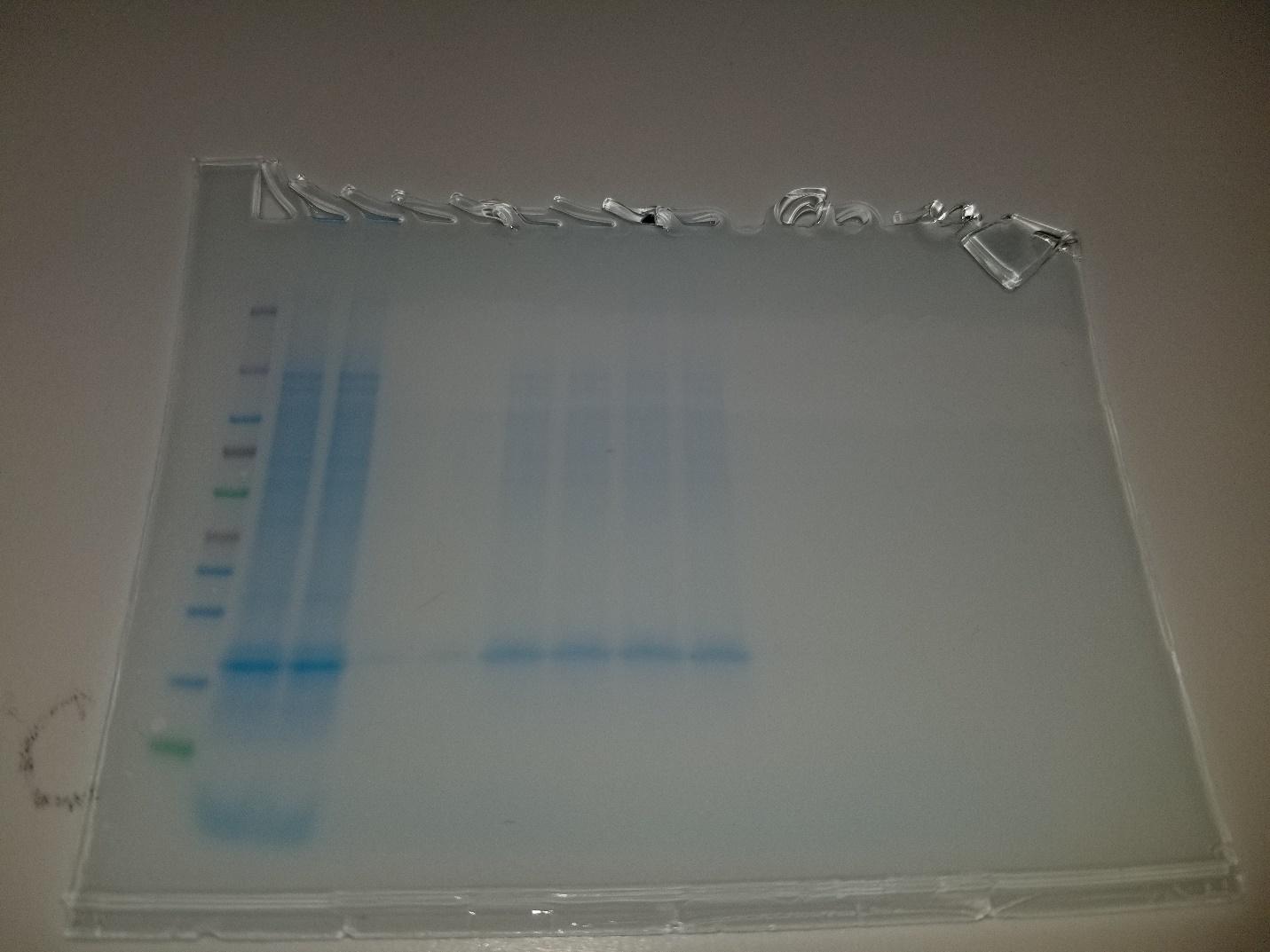


1. Niger protein extract

Control

PECO

**Fig. S9.** Electrophoresis gel image of *A. niger* protein extract degradation assay (15 min).


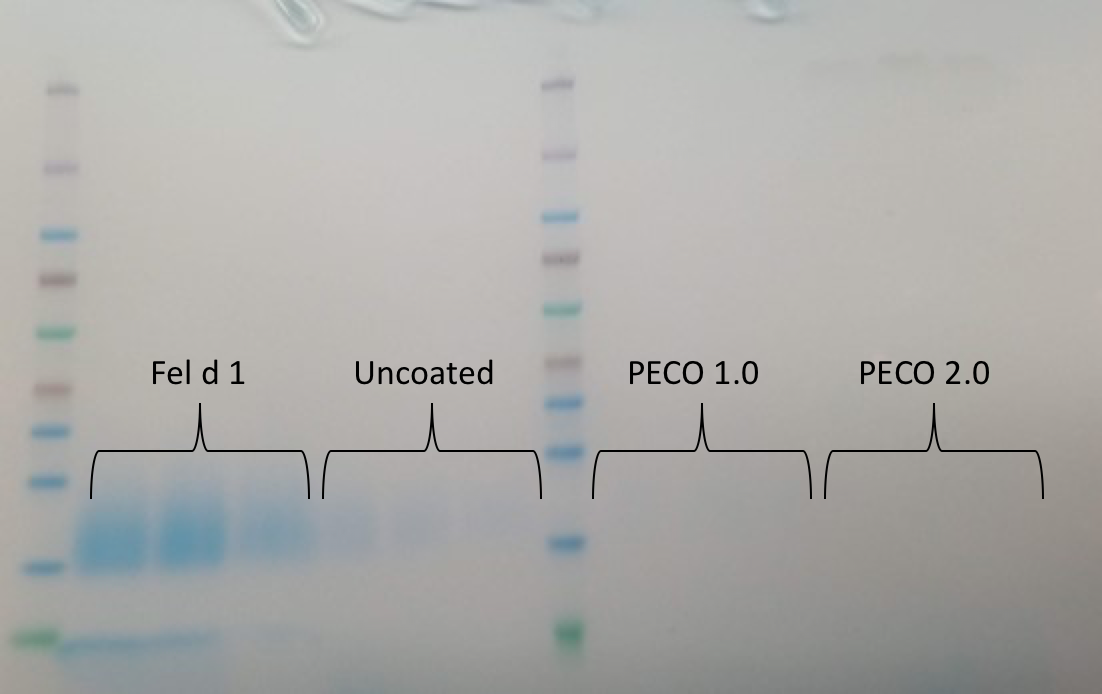


**Fig. S10.** Electrophoresis gel image of pure Fel d1 degradation assay.


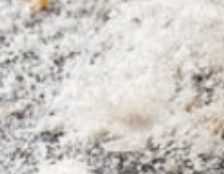


Extraction

Proteins including Feld 1

Peptides

Digestion

LC-MS

Data Analysis

Separation

**Fig. S11.** Proteomics with Cat Dander Extract.
